# O-GlcNAcylation at the tumor–immune interface: a metabolic post-translational code driving immune evasion and therapy resistance in cancer

**DOI:** 10.3389/fimmu.2026.1854952

**Published:** 2026-07-22

**Authors:** Shanshan Liu, Peng Zhan, WenJie Tian, Jinning Gu

**Affiliations:** 1 Department of General Medicine, the Second Hospital of Jilin University, Changchun, China; 2 Department of Urology, the Second Hospital of Jilin University, Changchun, China

**Keywords:** cancer metabolism, immune evasion, O-GlcNAc transferase, O-GlcNAcylation, therapeutic resistance

## Abstract

Protein O-GlcNAcylation has evolved from a metabolic curiosity into a master post-translational modification that enables cancer cells to translate nutrient availability into coordinated programs of proliferation, therapy resistance, and immune evasion. In this review, we argue that hyper-O-GlcNAcylation is not merely a passive consequence of the Warburg effect but an actively maintained stress-adaptive state that drives malignancy through parallel substrate-selective circuits rather than a single unified axis. We synthesize recent advances in four interconnected dimensions. Metabolically, OGT integrates glucose, lipid, and nucleotide metabolism by modifying key rate-limiting enzymes and is regulated by lineage-specific mechanisms. O-GlcNAcylation modulates responses to chemotherapy and radiotherapy, reinforces DNA damage repair, and controls senescence. Immunologically, it promotes tumor immune evasion by stabilizing PD-L1, reprogramming macrophages, suppressing NK and T cell function, and modulating cGAS–STING signaling, thereby influencing checkpoint blockade efficacy. In the tumor microenvironment, O-GlcNAc signaling remodels the extracellular matrix, drives angiogenesis, and maintains cancer stemness and mechanoadaptation. Collectively, we propose that substrate-selective O-GlcNAc circuits, rather than global OGT activity, are the key drivers of context-dependent malignancy. This framework highlights opportunities for developing next-generation low-toxicity therapeutics, including substrate-selective inhibitors and PROTAC degraders, in rational combinations with chemotherapy, radiotherapy, and immunotherapy.

## 1 Introduction

Protein post-translational modifications (PTMs) are covalent alterations that occur during or after protein synthesis and profoundly impact protein functions (1, 2). Among the more than 300 identified PTMs (3), glycosylation is one of the most diverse and abundant, encompassing N-linked, O-linked, C-linked, and glycosylphosphatidylinositol (GPI)-anchored subtypes (4, 5). Aberrant glycosylation is now recognized as a hallmark of malignancy that shapes tumor proliferation, metastasis, and immune evasion across virtually every cancer type (6, 7). Within the O-linked class, O-GlcNAcylation, which involves the addition of a single N-acetylglucosamine residue to serine and threonine residues of cytoplasmic, nuclear, or mitochondrial proteins (8), has emerged as a particularly consequential regulator of oncogenic signaling (9, 10). Unlike conventional protein glycosylation, O-GlcNAcylation is dynamic and reversible. O-GlcNAcylation is highly responsive to a wide range of extrinsic stimuli, including osmotic, oxidative, hyperthermic, and genotoxic stress (11, 12). The dynamic cycling of this modification is reversibly regulated by a single pair of antagonistic enzymes, O-GlcNAc transferase (OGT), which adds the sugar moiety, and O-GlcNAcase (OGA), which removes it, in a manner that is highly responsive to nutrient availability and cellular stress (11). This nutrient-sensitive PTM thus serves as a rapid molecular rheostat linking cellular metabolism to signaling, transcription, and stress responses (13).

The Warburg effect drives elevated hexosamine biosynthesis pathway (HBP) flux and global hyper-O-GlcNAcylation, a feature documented largely by pan-O-GlcNAc immunostaining across breast, prostate, lung, colorectal, liver, and hematologic malignancies (10, 14–16). O-GlcNAc transferase (OGT) is concurrently upregulated in virtually every tumor examined, and its silencing suppresses transformed phenotypes (17, 18). Mechanistically, O-GlcNAcylation regulates the core hallmarks of cancer, including proliferation, survival, invasion, metastasis, energy metabolism, and epigenetic reprogramming (9, 19–23). Specific substrates couple HBP activity to oncogenic output: O-GlcNAcylation of phosphofructokinase 1 (PFK1) at Ser529 diverts glycolytic intermediates into the pentose phosphate pathway (24), malate dehydrogenase 1 (MDH1) at Ser189 sustains glutamine catabolism in pancreatic cancer (25), and STAT5 drives oncogenic transcription in myeloid malignancies (26). The HBP–OGT axis further promotes tumor immune evasion by stabilizing programmed death ligand 1 (PD-L1). O-GlcNAcylation of the endosomal sorting component HGS impairs its interaction with PD-L1 and blocks lysosomal degradation of PD-L1 (27), whereas HBP hyperactivation upregulates PD-L1 translation by bypassing signal peptide-mediated elongation arrest (28), collectively licensing checkpoint-mediated immune evasion. Hyperglycemia-driven O-GlcNAcylation induces alternative (M2) macrophage polarization in the tumor microenvironment, reinforcing immunosuppression (29).

Despite significant advances, the mechanisms by which substrate-selective O-GlcNAc circuits, rather than a unified OGT–cancer axis, orchestrate metabolic reprogramming, therapy resistance, and immune evasion across different tumor lineages remain poorly understood. A methodological caveat must be stated at the outset, because it conditions how the entire literature below should be interpreted. The evidence for “global” hyper-O-GlcNAcylation across tumor types, together with most of the prognostic correlations discussed in later sections, rests predominantly on pan-O-GlcNAc detection using the monoclonal antibodies RL2 and CTD110.6. By design, these reagents integrate signals across thousands of substrates and therefore cannot distinguish oncogenic from tumor-suppressive site-specific modifications. More fundamentally, CTD110.6 is now known to cross-react with terminal β-N-acetylglucosamine on complex N-linked glycans, such that a portion of the “O-GlcNAc” signal recorded in bulk immunoblotting or immunohistochemistry may not reflect the intracellular modification (30, 31). Therefore, we interpreted bulk-level associations as hypothesis-generating tissue signatures rather than mechanistic evidence. Throughout this review, we deliberately focused on studies that resolve O-GlcNAcylation of defined substrates and modification sites. This distinction is not merely technical; it is foundational to the central thesis developed below, that substrate-selective O-GlcNAc circuits, rather than bulk OGT activity, drive context-dependent cancers. We revisit its translational consequences in detail in the Limitations and Unresolved Questions section. Critical questions remain regarding lineage-specific OGT regulation, O-GlcNAc–phosphorylation crosstalk, and the predictive value of substrate-resolved versus global O-GlcNAc profiles for therapeutic vulnerability. Addressing these gaps is essential for advancing O-GlcNAc biology toward substrate-directed and context-aware therapies. Here, we present a unifying framework in which hyper-O-GlcNAcylation acts as an actively maintained stress-adaptive state that drives malignancy through four interconnected axes: metabolic integration, therapeutic resistance, immune evasion, and tumor microenvironment remodeling. We also outline how this perspective can inform the design of next-generation O-GlcNAc-targeted interventions. Although O-GlcNAcylation in cancer has been reviewed previously, covering its cancer-associated inflammation (32) and immunotherapy links (33) and its role in therapy resistance (34), these accounts largely catalogue functions cancer-by-cancer and treat global OGT activity as broadly oncogenic. Here we instead advance a unifying, falsifiable convergence framework centered on substrate-selective, site-specific circuits, integrate the most recent (2024–2026) site-resolved and immune cell-intrinsic studies, and add an explicit translational lens (prognostic vs predictive biomarkers, evidence grading, and CNS-safety constraints).

## 2 O-GlcNAcylation machinery and hexosamine biosynthesis in cancer: a metabolic primer

The HBP end-product UDP-GlcNAc acts as a nutrient rheostat, and its elevation in tumor cells drives the coordinated O-GlcNAcylation of the metabolic machinery and OGT itself. In non-small cell lung cancer (NSCLC), glucose upregulates CARM1, which methylates OGT at arginine 348 to recruit the deubiquitinase USP9X, stabilizing OGT, and amplifying global O-GlcNAcylation to drive glycolysis, c-Myc induction, and tumor growth (35). Glycolytic enzymes are direct O-GlcNAc targets. In colon cancer, phosphoglycerate kinase 1 (PGK1) is O-GlcNAcylated at T255 to enhance lactate production and translocate into mitochondria, where it functions as a kinase that inhibits the pyruvate dehydrogenase (PDH) complex and suppresses oxidative phosphorylation (36). In NSCLC, hexokinase 2 (HK2) O-GlcNAcylation preserves the mitochondrial membrane potential, suppresses ROS, and promotes proliferation and invasion (37). Together, these circuits establish a glucose → CARM1 → OGT → glycolytic enzyme axis that constructs the metabolic ground state for downstream oncogenic programs.

Beyond glycolysis, O-GlcNAcylation acts as a multi-input integrator, converging on rate-limiting enzymes across orthogonal metabolic streams (modification sites in **Table 1**). In the lipogenic stream, it modifies ATP-citrate lyase (ACLY) to enable CoA binding and *de novo* lipogenesis, independent of EGF-driven phosphorylation (38). In nucleotide metabolism, it drives hexamer formation of phosphoribosyl pyrophosphate synthetase 1 (PRPS1), relieving feedback inhibition and AMPK restraint to boost purine, pyrimidine, and NAD synthesis and to confer chemoradiotherapy resistance in lung cancer (39). In glutamine catabolism, the modification of malate dehydrogenase 1 (MDH1) sustains the redox-supporting pathway of KRAS-mutant pancreatic cancer (25). The same logic extends to systemic and tumor-suppressive inputs: dietary fructose raises microbiota-derived acetate to elevate hepatic UDP-GlcNAc and hyper-O-GlcNAcylate eEF1A1 in hepatocellular carcinoma (40), while glucose-dependent O-GlcNAcylation of CK2 promotes CRL4^COP1-mediated p53 degradation, bypassing the p53 checkpoint without mutation (41). These parallel, substrate-selective circuits, rather than a single axis, translate the carbon, nitrogen, and lipid statuses into coherent oncogenic outputs.

**Table 1:** Substrate-specific O-GlcNAcylation in cancer metabolism.

Target/enzyme	Modification & site	Cancer type/model	Key findings	Evidence level	Ref.
PGK1	T255 O-GlcNAcylation	Colon cancer	Activates PGK1, enhances lactate production, translocates PGK1 to mitochondria where it inhibits PDH complex; coordinates glycolysis & TCA cycle to promote tumor growth.	Mouse *in vivo*	(36)
HK2	O-GlcNAcylation (site not specified)	Non-small cell lung cancer (NSCLC)	Promotes proliferation, migration and invasion; modulates mitochondrial dynamics and alleviates mitochondrial damage, enhancing tumor growth *in vivo*.	*In vitro*	(37)
OGT (via CARM1)	R348 arginine methylation stabilizing OGT	NSCLC	CARM1-mediated methylation of OGT recruits USP9X, stabilizes OGT, increases global O-GlcNAcylation and c-Myc, promoting glycolysis and proliferation.	Mouse *in vivo*	(35)
ACLY	S979 O-GlcNAcylation	Multiple tumor types (xenograft models)	Required for CoA binding and ACLY activity; couples glucose supply to *de novo* fatty acid synthesis and rapid tumor cell proliferation, independent of S455 phosphorylation.	Mouse *in vivo*	(38)
PRPS1	O-GlcNAcylation by OGT	Lung cancer	Triggers PRPS1 hexamer formation, relieves feedback inhibition, blocks AMPK-mediated phosphorylation; boosts *de novo* nucleotide & NAD synthesis and confers chemoradiotherapy resistance.	Mouse *in vivo*	(39)
eEF1A1 (global O-GlcNAcylation)	Hyper-O-GlcNAcylation via UDP-GlcNAc elevation	Hepatocellular carcinoma (HCC)	High dietary fructose → microbiota-derived acetate elevates glutamine/UDP-GlcNAc → hyper-O-GlcNAcylation of eEF1A1, driving HCC progression.	Mouse *in vivo*	(40)
CK2 (→ CRL4^COP1–p53 axis)	Glucose-dependent CK2 O-GlcNAcylation	Mammary tumorigenesis (PyMT model)	Blocks CK2 phosphorylation of CSN2, assembles CRL4^COP1 E3 ligase to degrade p53, derepresses glycolytic enzymes; mutation-independent p53 bypass in hyperglycemia-driven cancer.	Mouse *in vivo*	(41)
MDH1	S189 O-GlcNAcylation	Pancreatic ductal adenocarcinoma (PDAC)	Stabilizes substrate-binding pocket; enhances MDH1 activity and glutamine catabolism, supporting PDAC growth under Kras mutation.	Mouse *in vivo*	(25)
OGT & ACLY (via PI3Kβ)	OGT T985 phosphorylation; ACLY T639/S667 O-GlcNAcylation	Glioblastoma	PI3Kβ acts as protein kinase, phosphorylates OGT to enhance O-GlcNAcylation; ACLY O-GlcNAcylation boosts acetyl-CoA, fatty acid synthesis and H3 acetylation.	Mouse *in vivo*	(42)
HIF-1α/GLUT1	O-GlcNAcylation regulates HIF-1α stability	Breast cancer (and multiple)	O-GlcNAcylation sustains HIF-1α (lowers α-KG, inhibits pVHL interaction), drives glycolysis via GLUT1; loss triggers ER stress/CHOP-mediated apoptosis.	Human/clinical	(20)
PKM2 (via OGA)	O-GlcNAcylation of PKM2 (inhibitory)	Multiple human cancers	OGA upregulated in cancers; its acetyltransferase activity facilitates PKM2 O-GlcNAcylation, inhibiting PKM2 catalytic activity and promoting aerobic glycolysis.	Mouse *in vivo*	(43)
PKM2 (via ULK1)	S333 phosphorylation antagonizes O-GlcNAcylation	Breast cancer	ULK1 phosphorylates PKM2-S333, blocking O-GlcNAcylation, promoting tetramer formation, reducing nuclear c-Myc; attenuates the Warburg effect and inhibits tumor growth.	Mouse *in vivo*	(44)

Evidence level denotes the highest level of experimental validation reached in the cited study: *In vitro* (cell-line or cell-free/biochemical systems only); Mouse *in vivo* (validation in an animal model, xenograft, syngeneic, orthotopic, or genetically engineered, including studies that additionally provide confirmatory human tissue staining); Human/clinical (patient cohorts in which the O-GlcNAc–related marker is correlated with survival, prognosis, or treatment response). Descriptive expression in human tumor tissue without an associated clinical-outcome correlation does not, by itself, raise a study to the Human/clinical level.

Hyper-O-GlcNAcylation is further amplified under stress and fine-tuned by reciprocal phosphorylation in response to stress. In glioblastoma, PI3Kβ, canonically a lipid kinase, functions as a protein kinase that phosphorylates OGT at T985 under high glucose conditions, enhancing OGT activity and driving ACLY O-GlcNAcylation for acetyl-CoA production, fatty acid synthesis, and histone H3 acetylation (42). Under hypoxic conditions, O-GlcNAcylation stabilizes HIF-1α by restraining α-ketoglutarate-dependent hydroxylation, preventing CHOP-driven ER stress apoptosis (20). A recurring yin-yang logic tunes flux on shared substrates: OGA is paradoxically upregulated in cancer, and its cryptic acetyltransferase activity facilitates OGT-mediated O-GlcNAcylation of PKM2 to inhibit tetramerization and drive the Warburg phenotype (43), whereas ULK1-mediated S333 phosphorylation antagonizes PKM2 O-GlcNAcylation to restore tetramer activity and suppress c-Myc-driven glycolysis (**Table 1**) (44). This reciprocal architecture establishes O-GlcNAcylation as a dynamically tunable node, a motif that recurs in the immune regulatory substrates discussed in Section 3.

## 3 O-GlcNAcylation in immune evasion and immunotherapy resistance

### 3.1 O-GlcNAcylation-dependent regulation of the PD-1/PD-L1 axis

**Figure 1:**
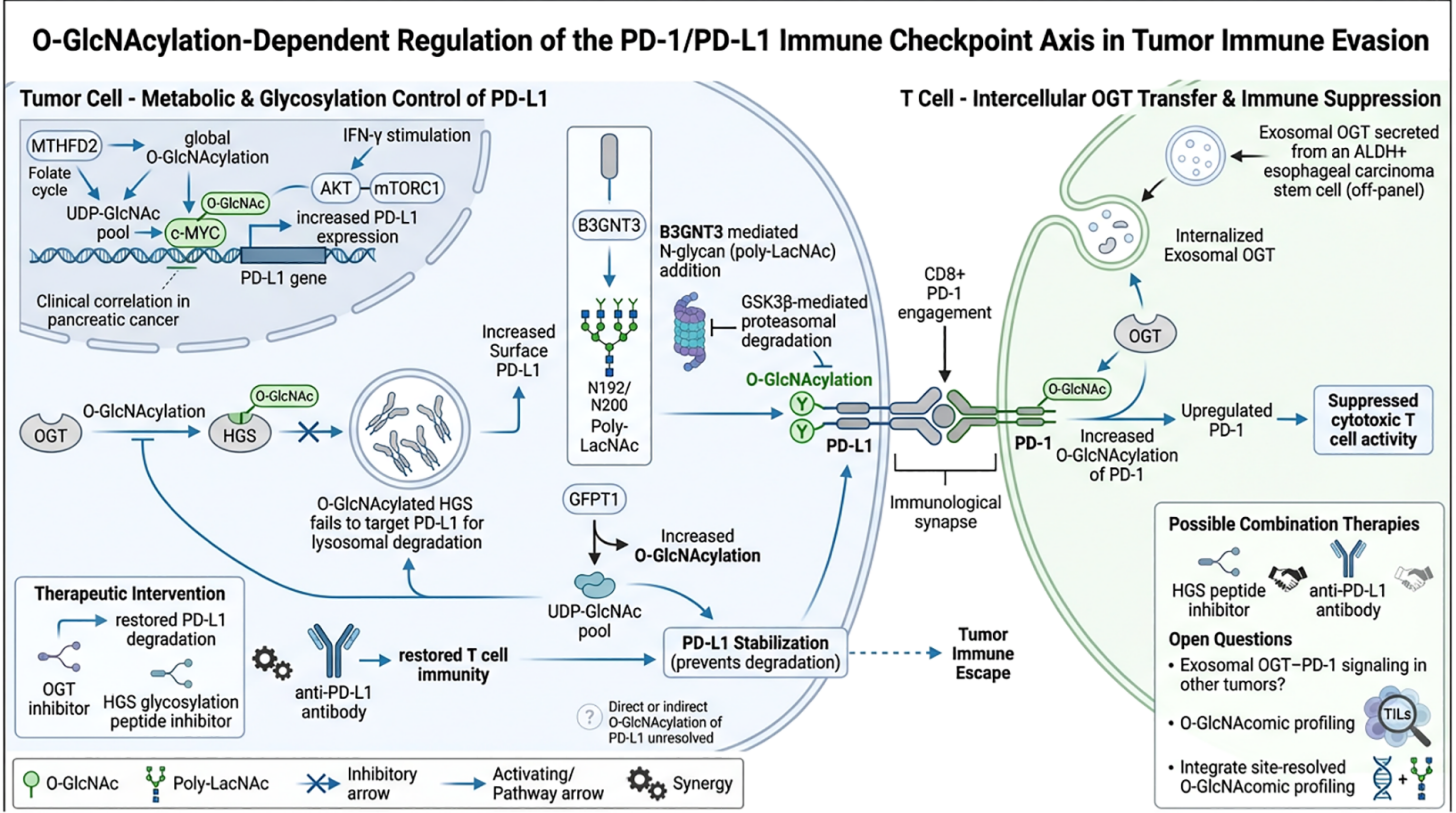
O-GlcNAcylation-dependent regulation of the PD-1/PD-L1 immune checkpoint axis in tumor immune evasion. O-GlcNAcylation modulates the PD-1/PD-L1 axis across multiple regulatory layers, including endosomal trafficking, metabolic control, transcriptional activation, and intercellular signaling. In tumor cells, O-GlcNAcylation impairs HGS-mediated lysosomal degradation and, together with GFPT1-driven hexosamine pathway flux and MTHFD2–c-MYC signaling, promotes PD-L1 stabilization and transcription. Additionally, exosomal transfer of OGT from ALDH^+^ cancer stem cells enhance PD-1 expression in CD8^+^ T cells, collectively reinforcing immune suppression and facilitating tumor immune evasion.

O-GlcNAcylation engages the PD-1/PD-L1 immune checkpoint axis at multiple regulatory levels, including lysosomal trafficking, protein stability, transcriptional control, and intercellular signalling, establishing the hexosamine pathway as a metabolic orchestrator of tumor immune evasion (**Figure 1**). Two mechanistically complementary studies have addressed post-translational PD-L1 stabilization. O-GlcNAcylation of HGS, a key endosomal sorting component, impairs its interaction with intracellular PD-L1, blocking lysosomal degradation and elevating surface PD-L1 levels to suppress T cell immunity (27). OGT inhibition or a competitive HGS glycosylation peptide inhibitor restored degradation and synergized with anti-PD-L1 antibody in immune-competent mouse models (27). Independently, GFPT1 upregulation in breast cancer elevates global O-GlcNAcylation and stabilizes PD-L1 through O-glycosylation, whereas GFPT1 silencing reduces PD-L1 levels and attenuates tumor immune escape *in vivo* (45). These studies converge on O-GlcNAc-dependent PD-L1 stabilization operating through spatially distinct nodes, endosomal sorting, and direct protein glycosylation, which could be targeted combinatorially. Notably, these O-GlcNAc-centric mechanisms operate in parallel to the far better-characterized N-linked glycosylation axis, in which B3GNT3-mediated poly-LacNAc modification of PD-L1 at N192/N200 shields it from GSK3β-driven proteasomal turnover and licenses PD-L1–PD-1 engagement in TNBC models (46). Whether the “O-glycosylation” invoked in the GFPT1 (45) reflects direct O-GlcNAcylation of PD-L1 itself or an indirect effect routed through N-glycan precursor supply remains unresolved, a caveat that tempers cross-tumor extrapolation.

A transcriptional layer was uncovered through MTHFD2, a folate cycle enzyme that sustains UDP-GlcNAc pools and global O-GlcNAcylation, stabilizing c-MYC to drive PD-L1 gene expression under IFN-γ stimulation via the AKT-mTORC1 pathway. O-GlcNAcylation levels are positively correlated with MTHFD2 and PD-L1 in patients with pancreatic cancer (47), providing direct clinical validation of this metabolic–epigenetic relay. Most strikingly, exosomal OGT secreted by ALDH+ esophageal carcinoma stem cells is taken up by CD8+ T cells, upregulating PD-1 and suppressing cytotoxic T cell activity (48), a directionally inverted intercellular mechanism that acts on the receptor rather than the ligand side of the checkpoint (**Table 2**).

**Table 2:** O-GlcNAcylation as a regulator of tumor immunity and immunotherapy resistance.

Cancer/Immune model	Immune outcome	O-GlcNAc target/regulator	Mechanistic findings	Evidence level	Ref.
Multiple tumor models (*in vitro* & immune-competent mice)	PD-L1/endosomal sorting	HGS (O-GlcNAcylated)	O-GlcNAcylation of HGS blocks its interaction with intracellular PD-L1, impairing lysosomal degradation and stabilizing surface PD-L1; OGT inhibition + anti-PD-L1 synergistically activates T-cell antitumor immunity	Mouse *in vivo*	(27)
Breast cancer (BC tissues, cell lines, xenograft)	PD-L1 stability	GFPT1 → HBP → PD-L1	GFPT1 elevates O-GlcNAc and stabilizes PD-L1 via O-glycosylation, accelerating BC immune escape; GFPT1 silencing reduces PD-L1 *in vivo*	Mouse *in vivo*	(45)
Esophageal carcinoma stem cells (ALDH+ ECSCs) & CD8+ T cells	PD-1 upregulation in CD8+ T cells	Exosomal OGT	OGT-loaded exosomes from ECSCs are taken up by CD8+ T cells and upregulate PD-1, protecting ECSCs from CD8+ T-cell killing	*In vitro*	(48)
Pancreatic cancer (and other tumors)	PD-L1 transcription	MTHFD2 → UDP-GlcNAc → cMYC O-GlcNAcylation	IFN-γ–AKT–mTORC1 induces MTHFD2, sustaining UDP-GlcNAc and global O-GlcNAcylation; O-GlcNAcylation stabilizes cMYC and drives PD-L1 transcription	Human/clinical	(47)
Tumor-associated macrophages (TAMs); GM-CSF–sufficient tumors	Protumoral TAM differentiation	EGR2 (Ser299) via OGT	O-GlcNAcylation of EGR2 at S299 enhances its binding to Trem2 and other myeloid genes, expanding C1QC+TREM2+MerTK+ TAMs and suppressing CD8+ T-cell cytotoxicity	Mouse *in vivo*	(49)
M2-like TAMs in tumor microenvironment	Cancer metastasis & chemoresistance	Cathepsin B (Ser210) via lysosomal OGT	Glucose flux through HBP drives O-GlcNAcylation of lysosomal Cathepsin B at S210, increasing its maturation and secretion in TME; correlates with chemo response in patients	Human/clinical	(50)
Hyperglycemic/diabetic hosts; CRC patients	M2 macrophage polarization	HBP → global O-GlcNAcylation in TAMs	Hyperglycemia increases HBP flux and O-GlcNAcylation in TAMs, skewing them to M2 phenotype and enabling immune evasion; M2 markers elevated in DM2 CRC patients	Human/clinical	(29)
Orthotopic HCC (C57BL/6) treated with Astragalus polysaccharide	NLRP3+ M1-like TAM polarization	OGT downregulation by APS	APS suppresses OGT and global O-GlcNAcylation in TAMs, enriching NLRP3+ pro-inflammatory TAMs (IL-6, IL-1β, TNFα, CXCL10), enhancing CD8+ T infiltration and reducing exhaustion	Mouse *in vivo*	(51)
NK92 cells; *in vivo* tumor models	NK cell cytotoxicity	OGT-ISS deletion; LRPPRC O-GlcNAc	Stable O-GlcNAcylation (OGA inhibition or OGT-ISS deletion) preserves NK cytotoxicity, downregulates TGF-β, upregulates type I IFN signaling and adhesion genes	Mouse *in vivo*	(52)
Primary NK cells; lymphoma xenograft	NK cytotoxic function	OGT (IL-2/IL-15 induced)	OSMI-1 inhibition of OGT reduces NKG2D, NKp44, TNFα, IFN-γ, perforin, granzyme B and impairs NK killing *in vivo*; OGA inhibition has no effect	Mouse *in vivo*	(53)
NK92 cells with sHLA-G1a/ILT-2	NK activating/inhibitory signals	O-GlcNAc on tyrosine kinase signaling	sHLA-G1a engages ILT-2, recruits SHP-1 and dephosphorylates kinases blocking MEK/ERK; O-GlcNAc modification participates in NK activating and inhibitory signaling	*In vitro*	(54)
Tumor-infiltrating Tregs (TIL-Tregs); mouse tumors & human data	Treg-mediated tumor immune tolerance	Glut3 → O-GlcNAcylation of c-Rel (NF-κB)	Glut3-driven glucose uptake elevates O-GlcNAcylation in TIL-Tregs; O-GlcNAcylation of c-Rel orchestrates a tumor-specific Treg gene program; Treg-Glut3 deletion abolishes tumor tolerance	Human/clinical	(55)
CD8+ T cells; adoptive cell therapy models	T-cell stemness vs exhaustion	β-catenin O-GlcNAcylation by OGT	D-mannose supplementation drives OGT-mediated O-GlcNAcylation of β-catenin, preserving Tcf7 and stem-like programming, enhancing anti-tumor efficacy of transferred T cells	Mouse *in vivo*	(56)
Uterine corpus endometrial cancer (UCEC)	PD-L1↑/MHC-I↓; ICB resistance	GR (Ser132) via OGT	OGT O-GlcNAcylates phosphorylated GR at S132, increasing PD-L1 and decreasing MHC-I; competitive peptide disrupts OGT–GR, restoring CD8+ T immunity	Mouse *in vivo*	(57)
Syngeneic & Apcmin CRC mouse models	cGAS-STING/type I IFN antitumor immunity	HCF-1 cleavage by OGT	OGT-mediated cleavage of HCF-1 maintains genomic stability; OGT loss induces genomic instability and cGAS-STING-dependent IFN-I and ISGs, enabling CD8+ T antitumor immunity	Mouse *in vivo*	(58)
Breast and lung cancer; anti-PD1 resistant tumors	Tumor macropinocytosis; MHC-II suppression	NRP1 O-GlcNAcylation by DHODH axis	DHODH sustains O-GlcNAcylation and membrane localization of NRP1, driving macropinocytosis; lysine/tryptophan accumulation glutarylates CIITA to repress MHC-II; DHODH inhibition reverses anti-PD1 resistance	Human/clinical	(59)
Breast cancer (tissues, cell lines, xenograft, PBMC co-culture)	CD39 stability; CD8+ T exhaustion	CD39 (Ser239) via GFPT1–OGT	GFPT1 interacts with OGT to mediate CD39 O-GlcNAcylation at S239, stabilizing CD39 and dampening CD8+ T cytotoxicity; GFPT1 knockdown enhances CD8+ infiltration and suppresses tumor growth	Mouse *in vivo*	(60)
Colorectal cancer (human tumors & models)	Aerobic glycolysis & PD-L1 stability	ENO1 (Thr19 & Ser249)	T19 O-GlcNAcylation forms active ENO1 dimers driving glycolysis; S249 O-GlcNAcylation blocks ENO1–STUB1 interaction stabilizing PD-L1; dual blockade synergizes with anti-PD-L1	Mouse *in vivo*	(61)

Evidence level = highest validation reached: *In vitro* (cells only); Mouse *in vivo* (animal models, ± confirmatory human staining); Human/clinical (patient cohorts with survival/prognosis/treatment-response correlation).

Future studies should establish whether exosomal OGT–PD-1 signalling operates across tumor types and whether HGS peptide inhibitors combined with checkpoint blockade produce durable responses. Site-resolved O-GlcNAc proteomic profiling of tumor-infiltrating lymphocytes from checkpoint-refractory patients provides a clinical framework for prioritizing these mechanistically distinct but potentially synergistic interventions.

### 3.2 Reprogramming of tumor-associated macrophages through O-GlcNAc signaling

**Figure 2:**
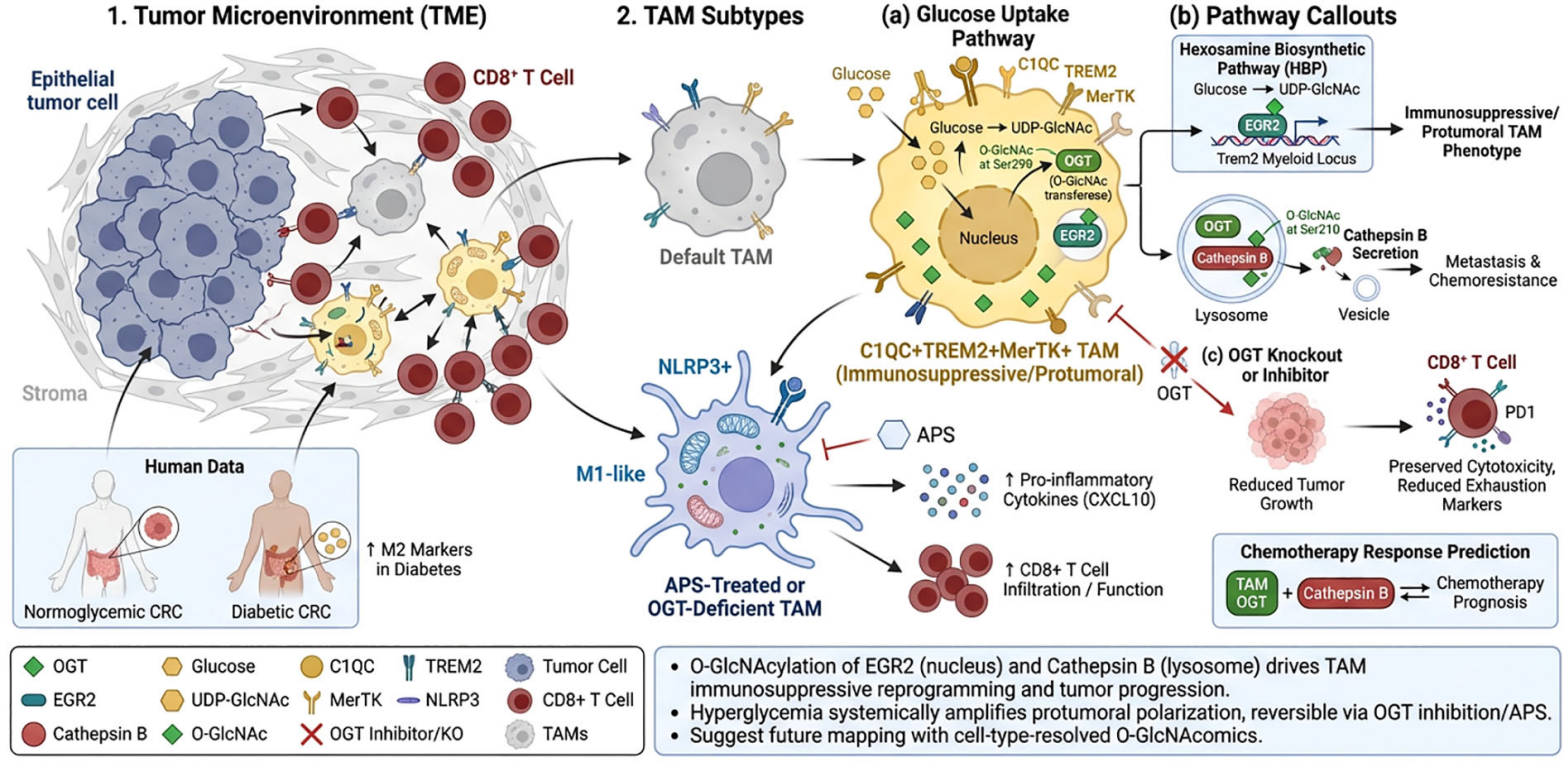
O-GlcNAc-driven reprogramming of tumor-associated macrophages in the tumor microenvironment. Glucose uptake fuels the hexosamine biosynthetic pathway (HBP) to elevate O-GlcNAcylation, which reprograms tumor-associated macrophages (TAMs) toward immunosuppressive, protumoral phenotypes. O-GlcNAcylation of EGR2 enhances its transcriptional activity to drive C1QC^+^TREM2^+^MerTK^+^ TAM differentiation, while lysosomal O-GlcNAcylation of Cathepsin B promotes its maturation and secretion, facilitating metastasis and chemoresistance. OGT deficiency or pharmacological inhibition suppresses tumor growth and restores CD8^+^ T cell function, whereas hyperglycemia amplifies TAM polarization toward M2-like states. Conversely, APS-mediated suppression of O-GlcNAcylation redirects TAMs toward NLRP3^+^ M1-like phenotypes, enhancing pro-inflammatory cytokine production and CD8^+^ T cell infiltration, highlighting OGT as a therapeutic and prognostic target.

Tumor-associated macrophages (TAMs) exhibit elevated glucose uptake relative to other TME constituents, and four studies have established that HBP-driven O-GlcNAcylation is the principal mechanism through which glucose metabolism reprograms TAMs into immunosuppressive, protumoral phenotypes (**Figure 2**). O-GlcNAcylation of EGR2 provides a transcriptional basis for protumoral TAM polarization. In mouse models, this modification enhances EGR2 binding to myeloid differentiation loci, such as Trem2, driving C1QC^+^TREM2^+^MerTK^+^ protumoral TAM differentiation (49). OGT deficiency in TAMs restricts tumor growth and preserves CD8+ T cell cytotoxic function while reducing exhaustion markers (49). A mechanistically distinct but functionally convergent effector was identified in M2-like TAMs, where glucose-driven O-GlcNAcylation of lysosomal Cathepsin B at Ser210 by a lysosome-localized OGT pool elevates mature Cathepsin B and drives its TME secretion, promoting metastasis and chemoresistance (50). TAM OGT levels and Cathepsin B expression correlated in human specimens and together predicted chemotherapy response and prognosis (50), providing direct clinical validation.

The hyperglycemic context of diabetes amplifies these effects systemically: elevated HBP flux skews macrophage polarization toward M2-like phenotypes, and M2 markers were specifically upregulated in TAMs from diabetic compared with normoglycemic colorectal cancer patients (29), implicating systemic metabolic disease as a driver of local immunosuppression and underscoring the clinical imperative for glycemic control in cancer therapy. Pharmacological reversal was demonstrated using astragalus polysaccharide (APS), which suppresses OGT and global O-GlcNAcylation in TAMs, redirecting polarization toward NLRP3+ M1-like phenotypes that secrete pro-inflammatory cytokines and CXCL10, enhancing CD8+ T cell infiltration, and reducing exhaustion in an orthotopic HCC model characterized by scRNA-seq (51). Nonetheless, these observations should be read against single-cell analyses that resolve TAMs into a continuum of transcriptional states rather than binary M1/M2 poles (62), raising the question of whether EGR2-driven differentiation and Cathepsin B secretion represent a unified O-GlcNAc-dependent program or context-restricted modules shaped by local cytokine milieux. The absence of side-by-side comparisons across tumor lineages tempers extrapolation from any single model to pan-cancer TAM biology.

Collectively, O-GlcNAc-modified EGR2 and Cathepsin B represent mechanistically distinct effectors of a conserved immunosuppressive program. Future studies should map the TAM O-GlcNAc proteome across human tumor types using cell-type-resolved O-GlcNAc proteomics and determine whether OGT inhibition synergizes with checkpoint blockade, particularly in hyperglycemic patients, in whom systemic HBP flux may render TAMs uniquely vulnerable (**Table 2**).

### 3.3 Dual role of O-GlcNAcylation in natural killer cell function

Natural killer (NK) cell cytotoxicity is metabolically licensed. O-GlcNAcylation, which is elevated by cytokine stimulation, is a permissive modification required for full NK effector function, and genetic stabilization offers a tractable strategy for NK-based immunotherapy. Two independent studies established that IL-2 and IL-15 stimulation increases O-GlcNAcylation in NK cells and that OSMI-1-mediated OGT inhibition comprehensively impairs cytotoxic capacity by reducing NKG2D, NKG2A, and NKp44 expression, diminishing granzyme B, perforin, granulysin, TNF-α, and IFN-γ, and abrogating tumor killing in a lymphoma xenograft model (53). Proteomics has identified LRPPRC as a key O-GlcNAc-modified NK cell regulator (52), although its mechanistic link to effector function requires further investigation. Critically, the studies diverge on enhancement: one study found that OGA inhibition or shRNA-mediated OGA knockdown did not augment cytotoxicity (53), whereas another demonstrated that Thiamet G and, more robustly, genetic deletion of the OGT intronic splicing silencer (ISS) stabilized O-GlcNAcylation, preserved potent cytotoxicity under TME-mimicking conditions, and enhanced *in vivo* tumor killing (52). Transcriptomic profiling of OGT-ISS-deleted NK92 cells revealed downregulated TGF-β and upregulated Type I interferon signalling, providing a molecular framework for TME suppression resistance (52). This discrepancy likely reflects the distinction between acute pharmacological and stable genetic O-GlcNAcylation elevation, which is a critical methodological caveat. This licensing logic broadly parallels observations in activated T cells, where TCR engagement drives a c-Myc–dependent HBP feed-forward loop in which O-GlcNAcylation stabilizes c-Myc and sustains glucose/glutamine flux to support clonal expansion (63). Whether analogous c-Myc or NFAT-centered circuits operate in cytokine-stimulated NK cells is untested, and the apparent NK-specific requirement for an LRPPRC-linked mitochondrial node (52) argues that effector-lineage-specific substrate maps, not T cell-derived frameworks, should guide rational O-GlcNAc engineering in NK cells. A recent study provides preliminary evidence that O-GlcNAcylation participates in both activating and inhibitory NK signalling downstream of the ILT-2 receptor (54), although the substrate identity and mechanistic details remain unresolved. Future studies should map the NK O-GlcNAc proteome under cytokine stimulation and TME suppression and identify the substrates that differentially govern cytotoxicity and exhaustion. Translating OGT-ISS deletion into primary NK cell manufacturing protocols would directly address the translational gap in O-GlcNAc-stabilized adoptive NK cell therapy (**Table 2**).

### 3.4 O-GlcNAc-mediated modulation of T cell differentiation and regulatory T cells

O-GlcNAcylation exerts opposing effects on T cell immunity depending on cellular identity; it sustains immunosuppressive Treg function at tumor sites while preserving cytotoxic CD8+ T cell stemness, a duality with fundamental implications for immunotherapy. In tumor-infiltrating regulatory T cells (TIL-Tregs), enhanced O-GlcNAcylation driven by Glut3-mediated glucose flux distinguishes TIL-Tregs from their systemic counterparts and orchestrates a tumor-specific transcriptional program (55). O-GlcNAcylation of the NF-κB subunit c-Rel underpins this process. Treg-specific Glut3 deletion abrogates tumor immune tolerance without disrupting systemic homeostasis, as validated in mouse models and human clinical data (55). This tumor-selective dependency positions Glut3 as an actionable target for Treg-specific immunotherapy without systemic autoimmune risk.

However, this relationship was inverted in CD8+ T cells. Single-cell metabolic profiling has identified diminished mannose metabolism as a hallmark of T cell dysfunction. D-mannose supplementation increased OGT-mediated O-GlcNAcylation of β-catenin, preserving Tcf7 expression and epigenetic stemness, while restricting exhaustion both *in vitro* and *in vivo* (56). Mannose-expanded T cells maintain stemness after extensive long-term expansion, enhancing the efficacy of adoptive therapy (56), reframing mannose as a metabolic input that decouples proliferation from exhaustion through O-GlcNAc-dependent β-catenin stabilization. These observations nuance an earlier paradigm in which HBP-driven O-GlcNAcylation was cast as a broadly proliferative signal in activated T cells via the stabilization of c-Myc and downstream nutrient uptake machinery (63). In contrast, the Treg and CD8+ mechanisms described here indicate that identical biochemical inputs can be channeled into opposing fate decisions, tolerance versus stemness, depending on which substrate (c-Rel versus β-catenin) dominates in a given subset, cautioning against the extrapolation of global OGT effects across T-cell lineages.

**Figure 3:**
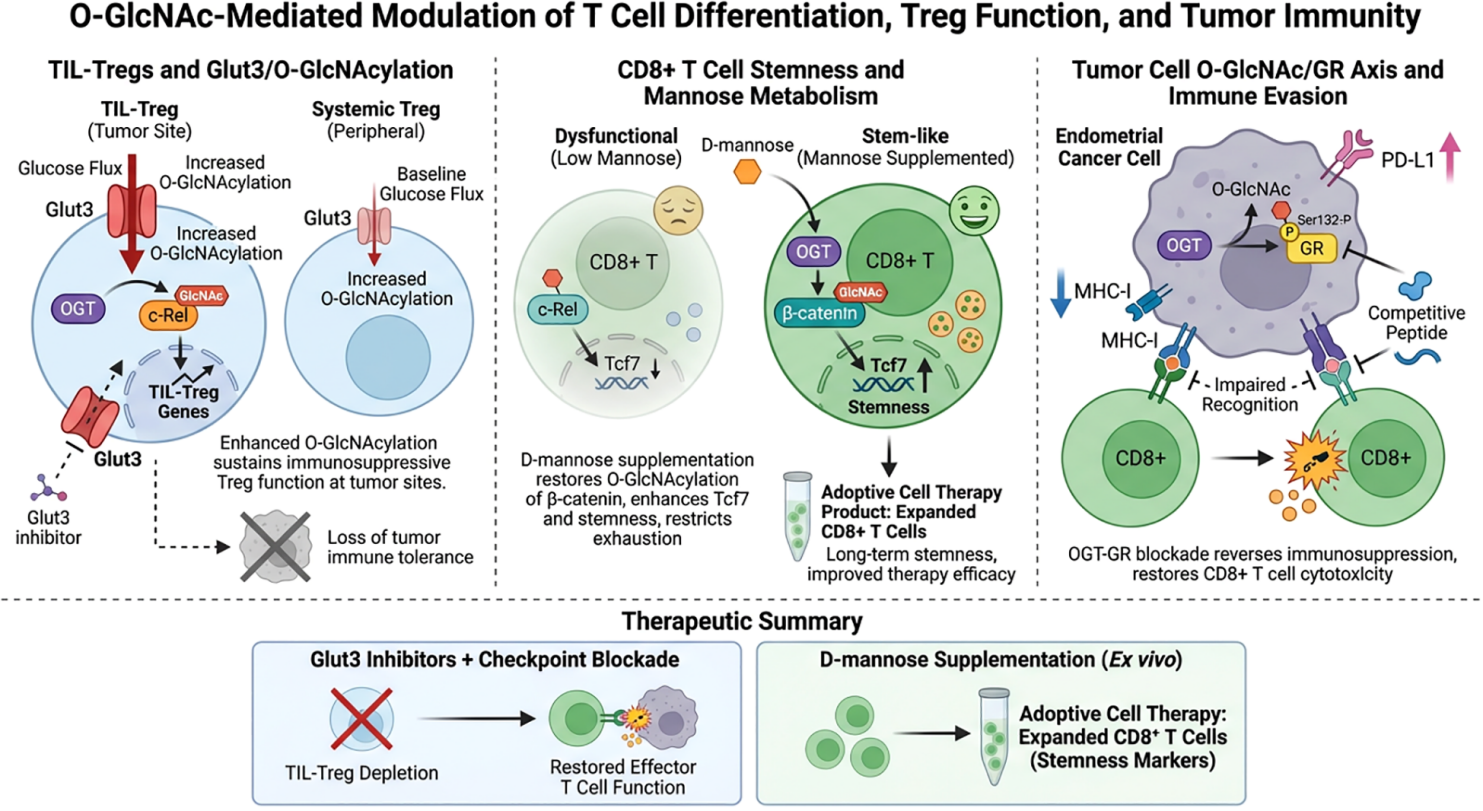
O-GlcNAc-mediated modulation of T cell differentiation, regulatory T cell function, and tumor immunity. O-GlcNAcylation exerts cell-type-specific and opposing effects on T cell immunity, sustaining immunosuppressive TIL-Treg function while preserving CD8^+^ T cell stemness. In tumor-infiltrating Tregs, Glut3-driven glucose flux enhances O-GlcNAcylation of c-Rel, promoting a tumor-specific transcriptional program that maintains immune tolerance. In contrast, D-mannose supplementation restores O-GlcNAcylation of β-catenin in CD8^+^ T cells, preserving Tcf7 expression, stemness, and resistance to exhaustion, thereby improving adoptive cell therapy efficacy. Additionally, tumor-intrinsic O-GlcNAcylation of the glucocorticoid receptor elevates PD-L1 and suppresses MHC-I, impairing CD8^+^ T cell recognition, while targeted disruption of the OGT–GR axis or Glut3 inhibition offers therapeutic opportunities to restore antitumor immunity.

A tumor-cell-intrinsic mechanism was revealed in endometrial cancer, where OGT O-GlcNAcylates the glucocorticoid receptor (GR) at Ser132, contingent on prior GR phosphorylation, elevating PD-L1 levels, and suppressing MHC-I to impair CD8+ T cell recognition (57). A competitive peptide disrupting the OGT–GR interface reversed this immunosuppressive state and restored CD8+ T cell killing *in vivo* (57), demonstrating that site-selective OGT substrate interference is a feasible therapeutic strategy for immunotherapy. Future studies should exploit these cell-type-resolved O-GlcNAc dependencies: Glut3 inhibitors combined with checkpoint blockade could simultaneously deplete TIL-Tregs and restore effector T cell function, while D-mannose supplementation during ex vivo expansion represents a metabolically straightforward approach to generating adoptive cell therapy products with durable stemness characteristics (**Figure 3**; **Table 2**).

### 3.5 O-GlcNAcylation as a regulator of cGAS–STING and innate immune pathways

O-GlcNAcylation restrains innate immune surveillance, and its inhibition has emerged as a way to simultaneously activate antitumor immunity and sensitize tumors to checkpoint blockade, although its action on the DNA-sensing machinery is bidirectional. The central tumor-intrinsic link runs through genomic stability: OGT-dependent cleavage of HCF-1 maintains genome integrity; therefore, OGT deletion triggers genomic instability, cGAS-dependent type I IFN production, and ISG induction, activating CD8^+^ T-cell antitumor immunity in syngeneic and Apc^Min^ colorectal cancer models (58). Deleting Cgas or Sting fully restored tumor growth in these OGT-null cells, confirming that the immune-activating effect is entirely cGAS–STING-dependent and recasting OGT inhibitors as innate-immune activators (58). Notably, this cancer cell-intrinsic suppressive role contrasts with antiviral studies in which OGT-mediated O-GlcNAcylation of STING at threonine 229 promotes its K63-linked ubiquitination and licenses type I interferon production during HSV-1 infection (64), and where SAMHD1 O-GlcNAcylation indirectly primes the same axis (65). The bottom line is directional context-dependence: O-GlcNAc restrains the tumor-suppressive output of DNA sensing while enabling its antiviral output, so the net effect of OGT inhibition will hinge on whether a given tumor depends on upstream instability-driven cGAS activation or on substrate-level STING regulation.

A structurally distinct axis was revealed through DHODH, which sustains O-GlcNAcylation and membrane localization of neuropilin-1 (NRP1) to drive tumor macropinocytosis (59). The resulting intracellular lysine and tryptophan accumulation promotes CIITA glutarylation, thereby repressing the expression of MHC-II. DHODH inhibition recruits immune cell infiltration and overcomes anti-PD-1 resistance *in vivo*, with a high DHODH/NRP1 ratio predicting poor prognosis in human breast and lung cancer (59). At the adaptive immune interface, GFPT1-driven OGT activity O-GlcNAcylates CD39 at Ser239, stabilizing this ectonucleotidase to generate an immunosuppressive adenosine-rich microenvironment. GFPT1 silencing enhances CD8+ T cell infiltration and cytotoxicity in breast cancer (60). Site-specific O-GlcNAcylation of ENO1 at Ser249 blocks the ENO1–STUB1 interaction, preventing STUB1-mediated PD-L1 degradation, orthogonally to ENO1-Thr19 glycosylation, which promotes glycolytic dimer formation (**Table 2**) (61).

Future studies should determine whether OGT inhibitor-induced cGAS–STING activation converts immunologically cold tumors into inflamed phenotypes and whether sequencing OGT inhibition before checkpoint blockade can maximize immune-priming. The ENO1 site-selective glycosylation model provides a template for bivalent inhibitors that simultaneously restrict both metabolic and immunosuppressive O-GlcNAc functions.

### 3.6 Cell-intrinsic O-GlcNAcylation across immune subsets: a divergent, lineage-specific logic

The preceding sections reveal that cell-intrinsic O-GlcNAcylation does not impose a single immunological direction; rather, its functional valence is lineage-specific and frequently opposite between effector and suppressor compartments. In cytotoxic effectors, O-GlcNAcylation is permissive for antitumor function: cytokine-induced O-GlcNAc is required for NK cell cytotoxicity, and its pharmacological removal collapses NKG2D expression and granzyme/perforin output (52, 53), while in CD8^+^ T cells, an O-GlcNAc-dependent β-catenin–Tcf7 axis preserves stemness and restrains exhaustion (56), and an HBP–c-Myc feed-forward loop sustains clonal expansion (63). In immunosuppressive and protumoral compartments, the same modification performs the opposite work: Glut3-driven O-GlcNAcylation of c-Rel sustains the tumor-specific suppressive program of intratumoral Tregs (55), and in tumor-associated macrophages, O-GlcNAcylation of EGR2 and lysosomal Cathepsin B, together with the GFPT2–YBX1–IL-18 axis, enforces M2-like, metastasis- and chemoresistance-promoting polarization (49, 50, 66), an effect amplified under hyperglycemia (29) and reversible by OGT suppression (51). Antigen-presenting cells occupy an intermediate, less-resolved position: in human monocyte-derived dendritic cells, OGT inhibition reshapes differentiation and maturation, altering AKT and MEK/ERK signaling, co-stimulatory and HLA-DR expression, and the IL-10/IL-6 balance, with downstream consequences for T-cell priming, the net tumor-relevant direction of which remains undefined (67).

This divergence has direct and underappreciated translational consequences. Because systemically delivered, non-selective OGT inhibition engages every immune compartment simultaneously, it is expected to disarm the effectors that mediate tumor rejection (NK and CD8^+^ T cells) while disabling the suppressors that enforce immune privilege (Tregs and TAMs). Therefore, the net effect on antitumor immunity cannot be deduced from tumor-intrinsic data and will depend on the dose, schedule, and dominant immune contexture of a given tumor. This logic tempers the optimistic reading of OGT inhibition as a uniform immune sensitizer and instead predicts a narrow, context-gated therapeutic window, reinforcing, from the immune side, the case for the cell-type-selective, substrate-directed, and tumor-restricted strategies developed in the Targeting O-GlcNAcylation section. It also exposes how much of the relevant landscape remains uncharted: cell-intrinsic O-GlcNAc biology in intratumoral dendritic cells and essentially all of its biology in myeloid-derived suppressor cells, B cells, neutrophils, and innate lymphoid cells, have not been mechanistically defined (68). Resolving the divergent, subset-specific dependencies summarized in **Table 3**, ideally through single-cell, cell-type-resolved O-GlcNAc profiling of human tumors–is therefore a prerequisite for predicting, rather than assuming, the immunological outcome of O-GlcNAc-targeted therapy.

**Table 3:** Lineage-specific effects of O-GlcNAcylation across immune cell subsets.

Immune subset	Principal O-GlcNAc substrate/node	Effect on the cell’s program	Net effect on antitumor immunity	Predicted effect of OGT inhibition	Ref.
NK cells	Cytokine-induced global O-GlcNAc; LRPPRC; OGT-ISS locus	Required for cytotoxicity (NKG2D, granzyme B, perforin, IFN-γ); genetic stabilization enhances killing	Pro-antitumor (effector-enabling)	Detrimental, impairs NK cytotoxicity	(52, 53)
CD8^+^ effector/memory T cells	β-catenin → Tcf7 (stemness); HBP–c-Myc loop	Preserves stemness, restrains exhaustion, supports clonal expansion	Pro-antitumor	Detrimental, erodes stemness/expansion	(56, 63)
Regulatory T cells (TIL-Treg)	c-Rel (via Glut3)	Sustains tumor-specific immunosuppressive program	Pro-tumor (immunosuppressive)	Beneficial, relieves Treg suppression (tumor-selective via Glut3)	(55)
Tumor-associated macrophages	EGR2-Ser299; Cathepsin B-Ser210; GFPT2–YBX1–IL-18	Drives M2-like/protumoral polarization, secretion, metastasis, chemoresistance	Pro-tumor	Beneficial, repolarizes toward M1	(29, 49–51, 66)
Dendritic cells	Not yet defined in tumors (moDC: AKT, MEK/ERK; CD80/CD86/HLA-DR; IL-10/IL-6)	Shapes differentiation/maturation and T-cell-priming capacity	Context-dependent/undetermined	Uncertain, alters maturation and priming	(67)

## 4 O-GlcNAcylation-mediated remodeling of the tumor microenvironment

### 4.1 O-GlcNAcylation of UGDH and extracellular matrix reprogramming

Extracellular matrix (ECM) remodeling is an underappreciated aspect of O-GlcNAc-mediated tumor progression. Two studies identified distinct substrates, UGDH and Hsp47, that converge on ECM composition and immune exclusion. O-GlcNAcylation of UGDH at Ser350 reinforces hydrogen bonding within the UDP-binding domain and enhances enzymatic activity, elevating hyaluronic acid (HA) synthesis (69). Molecular dynamics simulations provided atomic-level support, and CyTOF analysis confirmed an inverse correlation between UGDH O-GlcNAcylation and CD8+ T cell infiltration (69). O-GlcNAcylated UGDH also exerts a non-metabolic function by binding to KPNA2, displacing STAT1, blocking nuclear translocation, suppressing CXCL10 transcription, and impairing T cell recruitment (69). Tumors expressing O-GlcNAcylation-deficient UGDH exhibited reduced xenograft growth and improved survival rates.

In colorectal cancer, O-GlcNAcylation of the collagen chaperone Hsp47 promotes maturation and secretion of type I collagen. (70). Kaempferol disrupts this by reducing UDP-GlcNAc levels, downregulating OGT, impairing Hsp47 localization, and blocking collagen secretion both *in vitro* and *in vivo* (70). O-GlcNAc-modified Hsp47 has been proposed as a predictive biomarker for the OGT–collagen axis in the kidney. Together, these studies establish O-GlcNAcylation as a dual ECM remodeler that operates through enzymatic enhancement and chaperone regulation. However, both findings should be interpreted against the established principle that the bulk of the tumor ECM, including fibrillar collagens and HA, is deposited and crosslinked by cancer-associated fibroblasts (CAFs) and other stromal cells rather than by epithelial tumor cells (71). Because neither study resolves the contribution of stromal OGT activity to overall matrix deposition, it remains unclear whether tumor-intrinsic UGDH and Hsp47 modifications constitute the quantitatively dominant source of immunosuppressive ECM or a fine-tuning overlay on a predominantly CAF-driven program, a distinction with direct implications for the clinical positioning of OGT inhibitors. Future studies should investigate whether UGDH-driven HA and Hsp47-driven collagen cooperate to create a unified immune-exclusion barrier and whether OGT inhibition combined with checkpoint blockade can dismantle this barrier in ECM-dense, immunologically cold tumors.

### 4.2 O-GlcNAc-dependent promotion of tumor angiogenesis

Recent studies have established O-GlcNAcylation as a pro-angiogenic signal converging on VEGF regulation via cell-autonomous and intercellular mechanisms. In hepatocellular carcinoma, FOXM1 upregulates HBP flux, elevating O-GlcNAcylation and promoting VEGF secretion to drive angiogenesis; OGT inhibition significantly impairs this response (72). FOXM1 and SPP1 have been identified as hub HBP genes that correlate with poor prognosis, positioning HBP expression as a clinically informative prognostic signature (72).

A mechanistically precise intercellular axis was uncovered in bladder cancer, where tumor cells transfer GFAT1 to endothelial cells (ECs) via small extracellular vesicles (sEVs), reprogramming EC glucose metabolism by increasing hexosamine biosynthetic pathway (HBP) flux and O-GlcNAcylation (73). O-GlcNAcylation of SerRS at Ser101 promotes ubiquitination and degradation, while impairing importin α5-mediated nuclear translocation (73). Intranuclear SerRS suppresses VEGF transcription by binding to the GC-rich proximal promoter; its loss de-represses VEGF and drives neovascularization in the retina. GFAT1 knockout in tumor cells abrogated SerRS O-GlcNAcylation in ECs and attenuated angiogenesis *in vivo*, whereas sEV-mediated GFAT1 delivery restored angiogenic activity in GFAT1-knockout mice (73). Together, these studies reveal that O-GlcNAcylation promotes angiogenesis via VEGF secretion and transcriptional de-repression, which are complementary outputs of the same metabolic pathway. Nonetheless, these findings should be interpreted in the context of the broader recognition that tumor angiogenesis is predominantly governed by hypoxia-driven HIF-1α–VEGF signalling and stromal cues from CAFs and tumor-associated macrophages (74), rather than by a single metabolic input. Whether HBP-driven O-GlcNAcylation acts as an independent pro-angiogenic axis or converges on canonical hypoxia signalling, for example, by stabilizing HIF-1α, remains untested in either model, and the apparent quantitative dominance of the FOXM1 and sEV-GFAT1 mechanisms may reflect model-specific vascular dependencies rather than a pan-tumor principle. Future studies should establish whether the sEV-GFAT1 axis operates in other vascularized solid tumors and whether SerRS O-GlcNAcylation at Ser101 is detectable in circulating ECs as a minimally invasive angiogenesis biomarker for these tumors.

### 4.3 Intercellular metabolic communication via O-GlcNAc signaling

O-GlcNAc signalling functions as a metabolic sensor that translates extracellular nutrient cues into oncogenic programs. Two studies have demonstrated distinct modes of microenvironment-driven O-GlcNAcylation during intercellular tumor–stromal communication. In nasopharyngeal carcinoma, hypoxic tumor cell-derived exosomes (H-exo) carrying low LKB1 remodel adjacent adipocytes by inhibiting the STK11/LKB1–AMPK axis and activating the hexosamine biosynthetic pathway (HBP) flux (75). O-GlcNAcylation of VPS4B promotes its interaction with ANXA5, triggering lipid droplet (LD) maturation and release; tumor cells then internalize these LDs via macropinocytosis to fuel progression (75). This bidirectional circuit, in which tumor-derived exosomes program stromal adipocytes to supply lipid cargo, represents O-GlcNAc-dependent metabolic symbiosis in the TME.

In a systemic context, diabetic hyperglycemia elevates HBP flux and O-GlcNAcylation in oral squamous cell carcinoma (OSCC), promoting proliferation, invasion, and lymph node metastasis via the PI3K/AKT/mTOR pathway (76). DM status correlated with advanced T stage, depth of invasion, and Ki-67 in patients with OSCC; OGT knockdown or OSMI-1 reversed these phenotypes (76), reinforcing systemic glucose excess as a druggable determinant of tumor behavior.

Together, these studies illustrate that O-GlcNAcylation links local hypoxia-exosomes and systemic hyperglycemia inputs to tumor progression. The hyperglycemia–O-GlcNAc axis defined here for OSCC parallels earlier observations in colorectal cancer, where patients with diabetes exhibit skewing of tumor-associated macrophages toward immunosuppressive M2-like phenotypes via elevated HBP flux (29), suggesting that systemic glucose excess may act concurrently on tumor-intrinsic and myeloid compartments rather than on a single cell type. Whether the adipocyte-centered VPS4B circuit is similarly amplified under hyperglycemic conditions or remains a hypoxia-restricted mechanism has not been tested, which is a gap that tempers the direct extrapolation of either study to broader metabolic disease contexts. Future studies should map O-GlcNAc proteomic changes in adipocyte–tumor cocultures under nutrient stress and determine whether glycemic control in patients with diabetes and cancer reduces tumor O-GlcNAcylation and improves treatment response, a clinically tractable hypothesis warranting prospective investigation.

### 4.4 O-GlcNAcylation in tumor stemness, mechanoadaptation, and cytokine regulation

**Figure 4:**
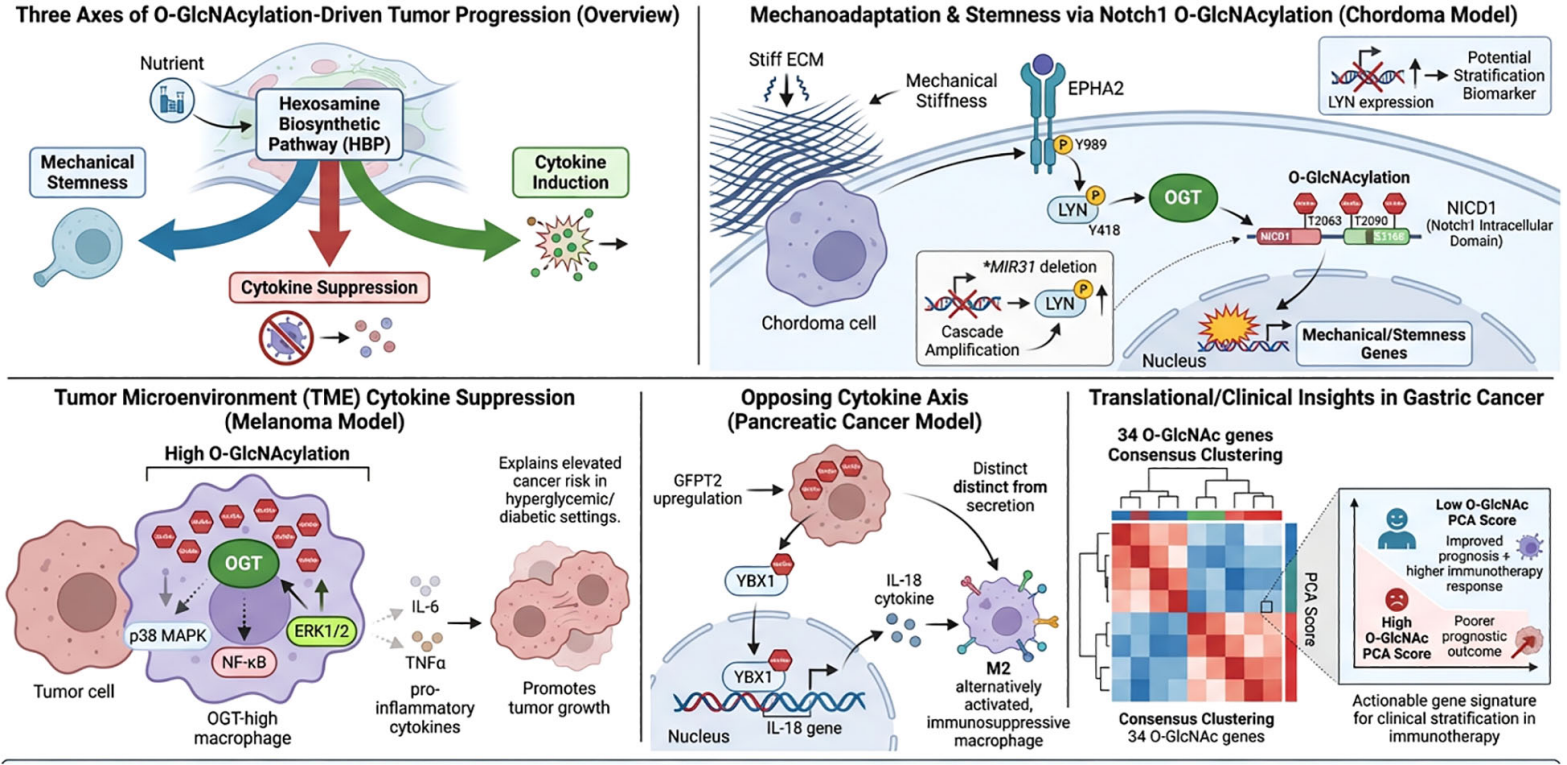
O-GlcNAcylation integrates tumor stemness, mechanoadaptation, and cytokine regulation. O-GlcNAcylation, driven by hexosamine biosynthetic pathway (HBP) flux, coordinates three distinct programs: mechanotransduction-linked stemness, suppression of pro-inflammatory cytokines, and transcription factor–mediated cytokine induction. Mechanistically, the EPHA2–LYN–OGT axis promotes site-specific O-GlcNAcylation of NICD1 to drive stemness gene expression, while opposing cytokine circuits, NF-κB suppression and YBX1-dependent IL-18 induction—reprogram macrophages toward immunosuppressive phenotypes, with clinical stratification supported by O-GlcNAc–related gene signatures in gastric cancer.

O-GlcNAcylation regulates tumor progression through three functionally distinct programs: mechanical stemness, cytokine suppression, and transcription factor-driven cytokine induction, all of which are unified by HBP flux as a shared metabolic sensor. The most mechanistically resolved axis involves Notch1 O-GlcNAcylation during chordoma recurrence. Stiff ECM drives ligand-independent EPHA2 phosphorylation, sequentially activating LYN kinase and OGT via phosphorylation at Y989 and Y418; enhanced OGT activity then induces NICD1 O-GlcNAcylation at T2063, T2090, and S2162, activating transcription of mechanical and stemness-related genes (77). MIR31 deletion amplifies this cascade by upregulating LYN expression and enhancing the perception of stiffness. MIR31 loss has been proposed as a patient stratification biomarker for Notch- or OGT-targeted therapy (77). This circuit represents a rare example of mechanotransduction directly and site-specifically coupled to glycosylation, with substantial therapeutic implications for recurrent chordomas. At the cytokine level, elevated TME O-GlcNAcylation, modelled using OGT-transgenic mice bearing B16 melanoma, suppresses p38 MAPK activity, upregulates ERK1/2 signalling, and downregulates macrophage NF-κB-driven pro-inflammatory cytokine production, thereby promoting tumor growth (78). This inverse relationship between TME O-GlcNAcylation and antitumor inflammation provides a mechanistic explanation for the elevated cancer morbidity observed in patients with diabetes and systemic hyperglycemia (78). A contrasting cytokine axis has been identified in pancreatic cancer, where GFPT2-mediated O-GlcNAcylation promotes the nuclear translocation of YBX1, which transcriptionally activates IL-18 and drives macrophage M2 polarization, creating an immunosuppressive TME (66). Recent studies converge on macrophage reprogramming through mechanistically orthogonal routes, NF-κB suppression versus YBX1-mediated IL-18 induction, a distinction with direct implications for therapeutic targeting in macrophage-rich tumors (66, 78). Translationally, consensus clustering of 34 O-GlcNAcylation-related genes in gastric cancer identified subgroups with divergent TME profiles. A principal component analysis (PCA)-derived prognostic score validated across five real-world cohorts demonstrated that patients with low scores had better prognosis and greater immunotherapy benefit (79), positioning O-GlcNAcylation gene signatures as a clinically actionable predictive framework (**Figure 4**). Future studies should establish whether the EPHA2–LYN–OGT mechanosensory axis operates in other stiff-ECM tumors, such as pancreatic and breast cancers, and prospectively validate the gastric cancer scoring system in immunotherapy trials to confirm its utility as a companion, diagnostic tool.

## 5 O-GlcNAcylation as a driver of chemotherapy resistance

### 5.1 HBP activation as a convergent chemo-stress response

Within the HBP-amplified state, O-GlcNAcylation drives resistance through discrete circuits best grouped by mechanism rather than drug class; the first class reprograms the DNA damage response (substrates and sites in **Table 4**). The clearest example of this is Erbin, a tumor suppressor that represses homologous recombination (HR). In 5-FU-resistant colorectal cancer, hyper-O-GlcNAcylation of Erbin promotes its degradation, lifting HR suppression, and enabling repair of 5-FU lesions. OGT silencing stabilizes Erbin and reverses resistance, both *in vitro* and *in vivo* (96). Three further substrates extend this theme: OGT stabilizes ADAR, driving A-to-I editing of DNA-repair transcripts in colorectal cancer (82); it modifies Polη to govern translesion synthesis (102); and in pancreatic cancer, it modifies RRM1 to maintain dNTP homeostasis, such that a non-modifiable RRM1 mutant induces replication stress and sensitizes cells to gemcitabine (87). A mechanistically distinct case is that of topoisomerase IIα (TOP2A). Rather than altering stability, O-GlcNAcylation directly enhances chromatin binding and catalytic activity, promoting adriamycin resistance in breast cancer cells and xenografts (91). Together, this class shows O-GlcNAc operating as a multi-layered regulator of genotoxic tolerance through degradation, transcript editing, translesion synthesis, nucleotide supply, and direct enzyme activation.

**Table 4:** O-GlcNAcylation as a driver of chemoresistance across human cancers.

Cancer model	Chemotherapy agent	Key regulator(s)	O-GlcNAc target/pathway	Mechanistic findings	Evidence level	Ref.
Lung carcinoma (H460, A549, H23)	Cisplatin (CDDP)	OGA inhibition → hyper-O-GlcNAcylation	p53/c-Myc	Hyper-O-GlcNAcylation promotes p53 ubiquitin-mediated proteasomal degradation and inhibits c-Myc ubiquitination, conferring CDDP apoptosis resistance independent of p53 status	*In vitro*	(16)
Lung cancer (*in vitro* & xenograft; human tumors)	Cisplatin	OGT (phosphorylated at Thr444 by AMPK)	NRF2 (Ser103)	O-GlcNAcylation of NRF2 at Ser103 blocks KEAP1 binding, stabilizes NRF2, lowers ROS and drives cisplatin resistance and malignancy	Mouse *in vivo*	(80)
SW620 metastatic colorectal cancer	Oxaliplatin (OXA)	OGT knockdown/OSMI-1; GFPT1 upregulated by OXA	Ribosomal proteins; DNA synthesome; glycolysis	OXA elevates O-GlcNAcylation and GFPT1; OGT inhibition sensitizes SW620 via apoptosis, cell-cycle arrest and ribosomal protein downregulation	*In vitro*	(81)
NSCLC H1299, HepG2, MCF-7 (*in vitro* & *in vivo*)	Cisplatin	OGT↑, OGA↑ (activity), AMPK↓, GFAT1 activation	Global O-GlcNAc/UDP-GlcNAc	Cisplatin elevates global O-GlcNAcylation by inhibiting AMPK→GFAT1, but OGT/OGA inhibition does not alter cisplatin sensitivity	Mouse *in vivo*	(82)
Colorectal cancer (HCT8, HCT116, 5FU-resistant)	5-Fluorouracil	OGT↑ in resistant cells	Erbin (Thr1070) → HR pathway	Hyper-O-GlcNAcylation of Erbin at Thr1070 drives its ubiquitin-dependent degradation, releasing homologous-recombination suppression and sustaining 5-FU resistance	::_	(83)
HT-29 (primary) & LoVo (metastatic) CRC	5-FU	OGT, O-GlcNAcylation	TYMS, TK1, TP	O-GlcNAcylation of TS/TK/TP differentially regulates 5-FU response: OGT knockdown reduces sensitivity in HT-29 but enhances it in LoVo	*In vitro*	(84)
HER2-negative breast cancer (*in vitro*, *in vivo*, patients)	5-FU/capecitabine + everolimus	Everolimus → OGT downregulation	TYMS	Everolimus suppresses OGT, reducing TYMS O-GlcNAcylation and destabilizing the TYMS homodimer via ubiquitin-independent proteasomal degradation, sensitizing tumors to 5-FU	Human/clinical	(85)
Pancreatic cancer	Gemcitabine	OGT, EIF3H deubiquitinase	METTL3 → HMGB1 (m6A-YTHDF2)	O-GlcNAcylation stabilizes METTL3 via EIF3H-mediated deubiquitination; METTL3 promotes HMGB1 degradation, suppresses ferroptosis and confers gemcitabine resistance	*In vitro*	(86)
Pancreatic cancer (PANC-1)	Gemcitabine	OGT	RRM1 (Thr734) → RRM2/RRM2B; TK1	RRM1 O-GlcNAcylation at T734 maintains dNTP balance and genomic stability; T734A mutation induces replication stress and enhances gemcitabine sensitivity	Mouse *in vivo*	(87)
Osteosarcoma	Methotrexate (MTX)	LncRNA EBLN3P → miR-200a-3p	OGT → EMT	EBLN3P sponges miR-200a-3p, upregulating OGT and driving EMT-mediated MTX resistance	*In vitro*	(88)
Triple-negative breast cancer (MDA-MB-231)	Cisplatin	OGT	c-Myc (Thr58)	OGT-mediated O-GlcNAcylation at c-Myc Thr58 stabilizes c-Myc, promoting colony formation and DDP resistance	*In vitro*	(89)
Breast cancer (MCF-7R, BT-549R; xenograft)	Adriamycin (doxorubicin)	OGT↑	MDM4 (Ser96) → glycolysis	OGT O-GlcNAcylates MDM4 at S96, promoting glycolysis and ADR resistance; OGT knockdown inhibits tumor growth *in vivo*	Mouse *in vivo*	(90)
Breast cancer cells & patient tissues; xenografts	Adriamycin	OGT	TOP2A (Ser1469)	O-GlcNAcylation of TOP2A at Ser1469 enhances chromatin DNA binding and catalytic activity, driving Adm resistance	Mouse *in vivo*	(91)
Hepatocellular carcinoma (HepG2; xenograft)	Doxorubicin (DOX) + OSMI-1	OGT inhibition (OSMI-1)	p53/mitochondrial Bcl-2	OSMI-1 synergistically enhances DOX-induced apoptosis via p53 and mitochondrial Bcl-2 pathways, reducing tumor growth *in vivo*	Mouse *in vivo*	(92)
Colorectal cancer	Oxaliplatin	KIAA1199 → O-GlcNAcylation	PARP1 (ER stress); SNAI1 (EMT)	KIAA1199 stabilizes PARP1 (reducing ER stress) and SNAI1 via O-GlcNAcylation, promoting OXA resistance and EMT	*In vitro*	(93)
Ovarian cancer	Cisplatin	OGT downregulation	SNAP-23 → SNARE (VAMP8/Stx4) → exosome release	Reduced O-GlcNAcylation of SNAP-23 promotes SNARE complex assembly and exosome-mediated cisplatin efflux, causing chemoresistance	*In vitro*	(94)
Breast cancer stem-like cells	Chemotherapy (general; CSC drug resistance)	OGT; ITCH (E3 ligase)	GATAD2B (C-terminal)/NuRD complex	C-terminal O-GlcNAcylation of GATAD2B blocks ITCH-mediated ubiquitination, sustaining CSC self-renewal and drug resistance	*In vitro*	(95)
Multiple cancer cell lines (established & primary)	Doxorubicin, camptothecin	HBP activation via AKT/XBP1	Global O-GlcNAcylation → survival signaling	Chemotherapy activates AKT/XBP1→HBP, elevating O-GlcNAcylation and survival pathways; suppression restores chemosensitivity	*In vitro*	(96)
Breast & colorectal cancer (xenograft)	Proteasome inhibitors	OGT–HCF-1 complex	NRF1 (proteasome bounce-back)	O-GlcNAcylation stabilizes NRF1, upregulating proteasome subunits; OGT inhibition sensitizes cancer cells to proteasome inhibitors	Mouse *in vivo*	(97)
Ovarian cancer (*in vitro* & xenograft)	Cisplatin	OGT downregulation	SNAP-29 → autophagy/SNARE	OGT down-regulation reduces SNAP-29 O-GlcNAcylation, promoting SNARE-driven autophagy and cisplatin resistance (paclitaxel unaffected)	Mouse *in vivo*	(98)
Bladder cancer (Gfpt1−/− mice; orthotopic)	Cisplatin/chemotherapy	GFPT1 → HBP/UDP-GlcNAc	NR3C1 (Thr299) → GPX4	GFPT1-driven HBP enhances NR3C1 Thr299 O-GlcNAcylation, stabilizing NR3C1 and transactivating GPX4 to suppress ferroptosis and cause resistance	Human/clinical	(99)
Prostate cancer (PC-3) vs WPMY-1	Doxorubicin, docetaxel + OSMI-1	OGT inhibition (OSMI-1)	Caspase-3/7 apoptosis	OSMI-1 synergistically lowers DOX IC50 in PC-3 cells, reducing OGT levels and increasing apoptosis	*In vitro*	(100)
NSCLC (chemoresistant xenograft)	Etoposide + Cisplatin (EP)	GFAT2 → HBP/UDP-GlcNAc	HSPD1 (Thr320) ← TRIM21	GFAT2 augments HSPD1 Thr320 O-GlcNAcylation, blocking TRIM21-mediated ubiquitination, stabilizing HSPD1 and activating anti-apoptotic signaling for EP resistance	Mouse *in vivo*	(101)

Evidence level = highest validation reached: *In vitro* (cells only); Mouse *in vivo* (animal models, ± confirmatory human staining); Human/clinical (patient cohorts with survival/prognosis/treatment-response correlation).

The most mechanistically compelling feedforward loop was delineated by Zhang et al., who showed that OGT O-GlcNAcylates NRF2 at Ser103 to sterically block KEAP1 binding and enhance NRF2 stability, nuclear localization, and antioxidant transcription; cisplatin itself activates AMPK, which phosphorylates OGT at Thr444 to strengthen the OGT–NRF2 interaction, a circuit in which the drug amplifies its own resistance mechanism, with elevated NRF2 O-GlcNAcylation confirmed in matched human lung cancer tissue (80). Extending this logic to oxaliplatin in colorectal cancer, OGT knockdown or OSMI-1 co-treatment in SW620 cells markedly enhanced apoptosis and cell cycle arrest, with proteomic profiling revealing the coordinated downregulation of ribosomal proteins and disruption of the DNA synthesome complex as additional resistance effectors (81). Parallel observations in etoposide/cisplatin-resistant NSCLC identified GFAT2 as a rate-limiting HBP driver whose upregulation sustains O-GlcNAcylation of HSPD1 at Thr320, blocking TRIM21-mediated ubiquitination and anti-apoptotic signalling (101). The generality of this rate-limiting HBP dependence is reinforced in AML, where enzymes of the hexosamine biosynthetic pathway are elevated in patient samples and where pharmacological GFAT inhibition or genetic/pharmacological OGT inhibition induces differentiation and apoptosis in AML cells while sparing normal cells, with tumor clearance in subcutaneous and circulating xenograft models and no signs of general toxicity (103). Collectively, platinum and topoisomerase poisons operate through at least three non-exclusive effector nodes: p53/c-Myc stability, NRF2-mediated antioxidant induction, and ribosomal/replicative integrity, each paradoxically reinforced by the chemotherapeutic stressor. The cisplatin–AMPK–OGT–NRF2 feedforward circuit is the most therapeutically actionable axis identified to date. Small-molecule disruptors of the OGT–NRF2 interface or site-specific blockade at NRF2-Ser103 can suppress adaptive resistance with greater selectivity than global HBP inhibition.

### 5.2 Substrate-selective resistance circuits: from DNA repair to oncoprotein stabilization and epitranscriptomic control

In the HBP-amplified state, O-GlcNAcylation operates through discrete circuits that can be grouped by mechanism rather than drug class. The first class reprograms the DNA damage response (DDR). In 5-FU-resistant colorectal cancer, Erbin, a tumorsuppressor thattrepressesg homologous recombination (HR), is hyper-O-GlcNAcylated at Thr1070, promoting its ubiquitin-mediated degradation, relieving HR suppression, and enabling efficient repair of 5-FUlesionsn.; OGT silencing stabilizes Erbin, restores HR repression, and reverses chemoresistance *in vitro* and *in vivo* (83). This Erbin-centered axis is complemented by OGT-driven stabilization of ADAR and consequent A-to-I editing of DNA damage repair transcripts in colorectal cancer (104), and by translation-synthesis control via Polη Thr457 O-GlcNAcylation (102), implicating O-GlcNAc as a multi-layered regulator of genotoxic tolerance. In pancreatic cancer, RRM1 O-GlcNAcylation at Thr734 maintains dNTP homeostasis: T734A knock-in impairs RRM2B binding, doubles dTTP through TK1 stabilization, and induces replication stress, which sensitizes PANC-1 cells to gemcitabine (87). In breast cancer, the most distinctive catalytic example is O-GlcNAcylation of topoisomerase IIα (TOP2A) at Ser1469, which enhances TOP2A chromatin binding and catalytic activity, directly modulating enzymatic function rather than stability, and promoting adriamycin resistance in cellular and xenograft models (91).

The second class stabilizes oncogenic transcription factors. In TNBC, c-Myc O-GlcNAcylation at Thr58 prevents proteasomal degradation and sustains oncogenic transcription, conferring chemoresistance that can be reversed by OGT inhibition (89). In adriamycin-resistant MCF-7R and BT-549R cells, O-GlcNAcylation of MDM4 at Ser96 upregulates glycolytic flux, whereas OGT knockdown reverses this phenotype and suppresses tumor growth *in vivo* (90). This illustrates that divergent substrate-level outcomes can converge to a shared pro-survival phenotype. Hyper-O-GlcNAcylation of the pioneer factor FOXA1 at Thr432/Ser441/Ser443 *destabilizes* FOXA1, silencing pro-apoptotic Bim and conferring bortezomib resistance, which can be reversed by the OGT inhibitor L01 (105). Parallel studies in HCC have demonstrated that OSMI-1 synergizes with doxorubicin to co-activate p53 and mitochondrial Bcl2 apoptotic pathways that neither agent engages alone (92). In the context of proteasome inhibitors, OGT–HCF-1-mediated O-GlcNAcylation of NRF1 drives proteasome subunit transcriptional bounce-back, with OGT inhibition sensitizing xenografts and OGT/proteasome co-expression validated in breast and colorectal meta-analyses (97).

A third-class act through enzyme-level regulation with context-dependent directionality is a principle with profound implications for clinical translation. Thymidylate synthase (TYMS), the primary 5-FU target, is stabilized by O-GlcNAcylation. Everolimus suppresses OGT, destabilizes TYMS homodimers through a ubiquitination-independent proteasomal pathway, and sensitizes HER2-negative breast cancer to 5-FU and capecitabine *in vitro*, *in vivo*, and in patient specimens (85). Very et al. extended this to colorectal cancer by identifying TYMS, thymidine kinase (TK), and thymidine phosphorylase (TP) as O-GlcNAcylated substrates, with a critical twist: OGT knockdown *reduces* 5-FU sensitivity in primary HT-29 cells by decreasing TK/TYMS yet *enhances* sensitivity in metastatic LoVo cells via elevated TP, demonstrating that the direction of O-GlcNAc effects is dictated by the stage-specific enzymatic landscape and cautioning against pan-OGT inhibition as a universal strategy (84). Beyond nucleotide enzymes, gemcitabine resistance in pancreatic cancer is further reinforced by O-GlcNAc-dependent stabilization of METTL3 through EIF3H-mediated deubiquitination, sustaining METTL3-driven HMGB1 degradation via the m6A–YTHDF2 axis, which suppresses ferroptosis (86). A clinically actionable parallel emerges in chemoresistant bladder cancer, where integrated metabolomic and O-GlcNAc proteomic profiling identified NR3C1 O-GlcNAcylation at Thr299 as a driver of GPX4 transcription, which suppresses ferroptosis. GFPT1 knockout induces ferroptosis and restores chemosensitivity, and GFPT1/NR3C1 levels in pre-chemotherapy biopsies serve as predictive biomarkers in training and validation cohorts (99). A final example is the EBLN3P lncRNA-mediated sponging of miR-200a-3p to upregulate OGT and drive EMT-linked methotrexate resistance in osteosarcoma, which extends this logic to non-coding regulatory input (88). Together, these circuits traverse enzyme stability, DNA repair, nucleotide metabolism, and epitranscriptomic control, and the context-reversed outcomes underscore why site-selective OGT inhibitors and O-GlcNAc proteomic patient stratification are clinical prerequisites rather than optional refinements.

### 5.3 Cellular plasticity, vesicular efflux, and context-reversed resistance

The third resistance layer operates through cell-state remodeling and vesicular trafficking, programs that extend beyond individual oncoprotein stabilization. In colorectal cancer, KIAA1199 exploits O-GlcNAcylation bifunctionally: it reduces ER stress to upregulate PARP1 and suppresses oxaliplatin-induced apoptosis, while simultaneously stabilizing the EMT driver SNAI1 (93). This dual output sits within a broader OGT/SNAI1 regulatory logic: SNAI1 is directly O-GlcNAcylated at Ser112, which blocks GSK3β-mediated phosphorylation and proteasomal turnover, reinforcing an EMT–stemness program that antagonizes p53-dependent apoptosis during chemotherapy (106, 107). The maintenance of cancer stem-like cells (CSC) constitutes a parallel axis. In breast cancer, C-terminal O-GlcNAcylation of GATAD2B, a NuRD chromatin-remodeling subunit, protects it from ITCH-mediated ubiquitination. Site-specific mutations abolish mammosphere formation, self-renewal factor expression, and drug resistance, establishing a causal link between site-specific glycosylation and tumor-initiating capacity (95).

Critical context-dependency is exemplified by ovarian cancer, where, in contrast to all preceding examples, loss rather than gain of O-GlcNAcylation confers resistance. OGT downregulation reduces SNAP-23 O-GlcNAcylation, promoting SNAP-23–VAMP8–Stx4 SNARE complex assembly and enhancing exosome-mediated cisplatin efflux (94). Reduced SNAP-29 O-GlcNAcylation facilitates SNARE-driven cytoprotective autophagy (98). OSMI-1 synergizes with doxorubicin to enhance caspase-3/7-mediated apoptosis in prostate cancer PC-3 cells (100), and isotope-coded photocleavable probes coupled with quantitative mass spectrometry have begun to resolve site-level stoichiometric differences between sorafenib-sensitive and -resistant liver cancer cells (108), providing a methodological foundation for substrate-resolved clinical profiling. The ovarian SNAP-23/29 axis, together with the HT-29/LoVo dichotomy discussed above, establishes that global OGT suppression is not uniformly safe; it can paradoxically promote resistance in contexts where specific substrate modification is protective. Collectively, O-GlcNAc cycling orchestrates resistance at three spatially distinct levels: nuclear transcription factor stability, chromatin remodeler integrity, and membrane-proximal vesicular efflux, and demands combinatorial strategies tuned to the dominant node in each tumor.

Three features of this resistance code are directly relevant to immune checkpoint combinations. First, the METTL3–HMGB1 and NR3C1–GPX4 axes converge to suppress ferroptosis, a cell death program whose release of damage-associated molecular patterns is increasingly recognized as a potent trigger of antitumor immunity. A third mechanistically deeper layer was recently defined in hepatocellular carcinoma, where O-GlcNAcylation functions as a direct redox sensor coupling oxidative stress to ferroptosis evasion. Reactive oxygen species oxidize OGT at Cys845 to activate the enzyme, which then O-GlcNAcylates the transcription factor FOXK2, driving its importin-α–dependent nuclear translocation and transcriptional upregulation of the cystine transporter SLC7A11 to restrain ferroptosis and confer chemoradiotherapy resistance (109). This ROS–oxidation–O-GlcNAcylation cascade is conceptually significant because it positions O-GlcNAcylation not merely as a downstream effector but as a sensor that converts a therapy-induced oxidative signal into a coordinated resistance response, an exemplar of the stress-adaptive, temporally coupled logic central to the convergence model proposed here. Therefore, blocking O-GlcNAc-driven ferroptosis may reduce the immunogenicity of chemotherapy. Second, Thr58 O-GlcNAcylation-dependent c-Myc stabilization directly feeds into transcriptional programs sustaining PD-L1 expression, providing a mechanistic link between chemoresistance and checkpoint evasion. Third, the directional reversibility of OGT effects suggests that global inhibitors are unlikely to uniformly synergize with immunotherapy and that substrate-selective strategies guided by tumor-specific O-GlcNAc proteomic profiling are required to realize chemo-immunotherapy combinations.

## 6 O- GlcNAcylation as a multi-layered regulator of DNA damage response and radiation sensitivity

### 6.1 O- GlcNAc-dependent amplification of DSB repair drives genotoxic and radiation resistance

O-GlcNAcylation directly regulates DNA double-strand break (DSB) repair through multiple substrates spanning both non-homologous end joining (NHEJ) and homologous recombination (HR) arms. The NHEJ scaffold XRCC4 is O-GlcNAcylated at Thr308, blocking TRIM21-mediated ubiquitination and elevating XRCC4 levels during DSB damage, thereby enhancing NHEJ fidelity and conferring resistance to DSB-inducing agents (110). In the HR arm, the end-resection factor And-1 is O-GlcNAcylated within minutes of ionizing radiation at Ser575 and Ser893 in its SepB domain, disrupting an intramolecular HMG–SepB interaction and freeing the HMG domain to bind DSBs and recruit CtIP (111). The And-1(2SA) mutants showed defective DSB accumulation, impaired RPA loading, and reduced Chk1 phosphorylation. Critically, in radioresistant colorectal cancer, the upregulation of OGT/And-1 O-GlcNAcylation is reversed by the FDA-approved bazedoxifene, providing a translatable resensitization strategy (111). MND1, a meiosis-specific HR factor aberrantly expressed in breast cancer, is O-GlcNAcylated at Thr121, which inhibits ubiquitination and maintains nuclear retention. The T121A mutation or OGT inhibition destabilizes MND1, sustains γH2AX elevation, and disrupts the MND1–HOP2 complex, thereby suppressing tumor growth *in vivo* (112).

These substrate-level findings sit within a broader chromatin framework: O-GlcNAcylation of H2AX at S139 and MDC1 restrains rather than potentiates γH2AX spreading (113), whereas H2B S112 GlcNAcylation recruits NBS1 to license both HR and NHEJ (114). The tension between such signal-dampening histone modifications and the unambiguously pro-repair stabilization of XRCC4, And-1, and MND1 indicates that O-GlcNAcylation operates as a context-dependent rheostat shaped by substrate stoichiometry and cell cycle phase, an essential caveat against extrapolating single-substrate vulnerabilities into pan-tumor sensitization strategies.

This rheostat logic extends into radiosensitivity through substrate-specific stabilization of pro-survival effectors. In glioblastoma, melanophilin (MLPH) is O-GlcNAcylated at Ser510, which blocks TRIM21-mediated degradation and enables MLPH-driven NF-κB activation. MLPH and its O-GlcNAcylation are elevated in recurrent post-irradiation GBM, establishing a self-reinforcing resistance loop that has been validated in xenografts (115). A mechanistically distinct axis operates in esophageal squamous cell carcinoma, where OGT O-GlcNAcylates RSK4 at Thr405, directly antagonizing GSK3β-mediated phosphorylation at adjacent Thr402/Ser406 and blocking FBXW7-dependent degradation (116). RSK4 stabilization enhances cancer stem cell properties and radioresistance, whereas OSMI-4 destabilizes RSK4 and radiosensitizes patient-derived xenografts and organoids (116), among the most clinically robust validations in this field. The RSK4 axis expands the mechanistic repertoire beyond ubiquitination blockade to inter-modification crosstalk at adjacent residues.

Counterintuitively, a retrospective cohort study of 216 NSCLC patients showed that high pretreatment tumor O-GlcNAc levels independently predicted favorable radiotherapy response (OR = 2.47; P = 0.005), superior progression-free survival (P = 0.049), and overall survival (P = 0.008), while glycemic parameters and SUVmax showed no association (117). This paradox likely reflects the divergence between bulk O-GlcNAcylation as a metabolic tissue signature and discrete effector-level resistance activity, and its resolution will require Site-resolved O-GlcNAc proteomic profiling in prospective cohorts rather than bulk measurements.

### 6.2 O-GlcNAcylation as a metabolic switch between therapy-induced senescence and apoptosis with immunological consequences

Therapy-induced senescence (TIS) arrests rather than eliminates cancer cells, enabling recurrence while sustaining a senescence-associated secretory phenotype (SASP) that reshapes the local immune microenvironment. Two complementary studies have established O-GlcNAcylation as a metabolic switch governing the senescence–apoptosis decision. In MCF7 xenografts, HBP-driven O-GlcNAcylation enhanced DSB repair and suppressed TIS. Exogenous GlcNAc or OGA targeting protects tumors from radiation, whereas OGT inhibition delays repair and elevates senescence *in vivo* (118), consistent with earlier mechanistic studies linking cancer metabolism to DNA repair and accelerated senescence (119). In colorectal cancer, low-dose SN38 or etoposide induced TIS in p53-proficient HCT116 and LS174T cells, alongside coordinated reductions in GFAT, OGT, and OGA. Further suppression via GFAT/OGT knockdown or OSMI-4 redirected the response from senescence to apoptosis, as validated in patient-derived tumoroids (120).

The immunological implications of this switch are significant. Senescent tumor cells actively shape the local immune microenvironment through SASP-mediated recruitment and polarization of myeloid populations and modulation of T cell infiltration. In contrast, apoptotic cell death can be immunogenically silent or stimulatory. Therefore, the O-GlcNAc–senescence axis directly intersects with anti-tumor immune surveillance. Pharmacological redirection of this decision by OGT inhibition may therefore not only eliminate the residual tumor reservoir but also reshape the immune contexture in ways that could synergize with checkpoint blockade, a hypothesis that warrants prospective testing in immune-competent models. Identifying the O-GlcNAcylated substrates that maintain the senescent state, likely chromatin remodelers and checkpoint effectors, and establishing their OGT dependence across tumor types, will be essential for designing senescence-to-apoptosis switching strategies coherent with the antitumor immune programs examined in subsequent sections.

## 7 A convergent mechanistic framework for O-GlcNAc–mediated therapeutic resistance

### 7.1 Convergent substrates and signaling hubs underlying cross-resistance

The four canonical arms of therapeutic failure–chemoresistance, radioresistance, immune evasion, and TME remodeling–have historically been studied as parallel escape routes governed by distinct effectors. Emerging evidence, however, indicates that O-GlcNAcylation functions as a unifying biochemical currency that mechanistically welds these arms together, such that tumors acquire multi-modal cross-resistance from shared substrates and convergent signaling hubs, rather than from independently evolved programs. We term this architecture the *convergence model*, in which a limited set of O-GlcNAc-modified node channels diverse therapeutic stresses into a coordinated molecular response.

The convergence model operates at three hierarchical levels: At the substrate level, individual proteins simultaneously execute functions across multiple resistance pathways. Enolase 1 is paradigmatic: dual site-specific glycosylation at Thr19 amplifies aerobic glycolysis, whereas modification at Ser249 stabilizes PD-L1, coupling metabolic reprogramming to immune escape within a single protein (61). NRF2 glycosylation at Ser103 escapes KEAP1-mediated degradation to concurrently dampen oxidative stress, drive malignancy, and confer cisplatin resistance in lung and ovarian cancers (80). c-Myc stabilization at Thr58 by OGT sustains chemoresistant transcriptional states across tumor types (16, 89), while within tumor-associated macrophages, glucose-fueled O-GlcNAcylation of lysosomal Cathepsin B couples stromal reprogramming to metastatic dissemination and chemoresistance (50). UGDH glycosylated at Ser350 further bridges hyaluronan-driven ECM remodeling with suppression of CXCL10-mediated CD8^+^ T-cell infiltration, linking TME architecture to immune exclusion (69).

At the pathway level, convergence intensifies through the shared signaling hubs. O-GlcNAcylation activates PI3K/Akt via DDX46 stabilization (121), amplifies Akt–NF-κB signaling to drive invasion and TME remodeling (122), and suppresses cGAS-STING–dependent type I interferon responses to enable immune evasion (58). These hubs are engaged across all four resistance arms, producing a highly interconnected network in which the disruption of any single node propagates phenotypic consequences across multiple therapeutic modalities.

At the temporal level, this crosstalk is acute and self-reinforcing. Cisplatin rapidly activates the hexosamine biosynthetic pathway through AMPK/GFAT1 modulation, elevating UDP-GlcNAc and global O-GlcNAcylation within hours (82, 96, 99), engaging all four arms simultaneously and establishing a feed-forward vicious cycle in which therapy itself fortifies every resistance arm. Mechanistic overlap extends to fate-switching decisions: O-GlcNAc inhibition redirects colon cancer cells from therapy-induced senescence to apoptosis (120), revealing a shared decision node bridging chemoresistance and radioresistance. Collectively, these convergent substrates, pathways, and temporal dynamics establish O-GlcNAcylation as a central molecular hub orchestrating pan-therapeutic resistance in cancer.

**Figure 5:**
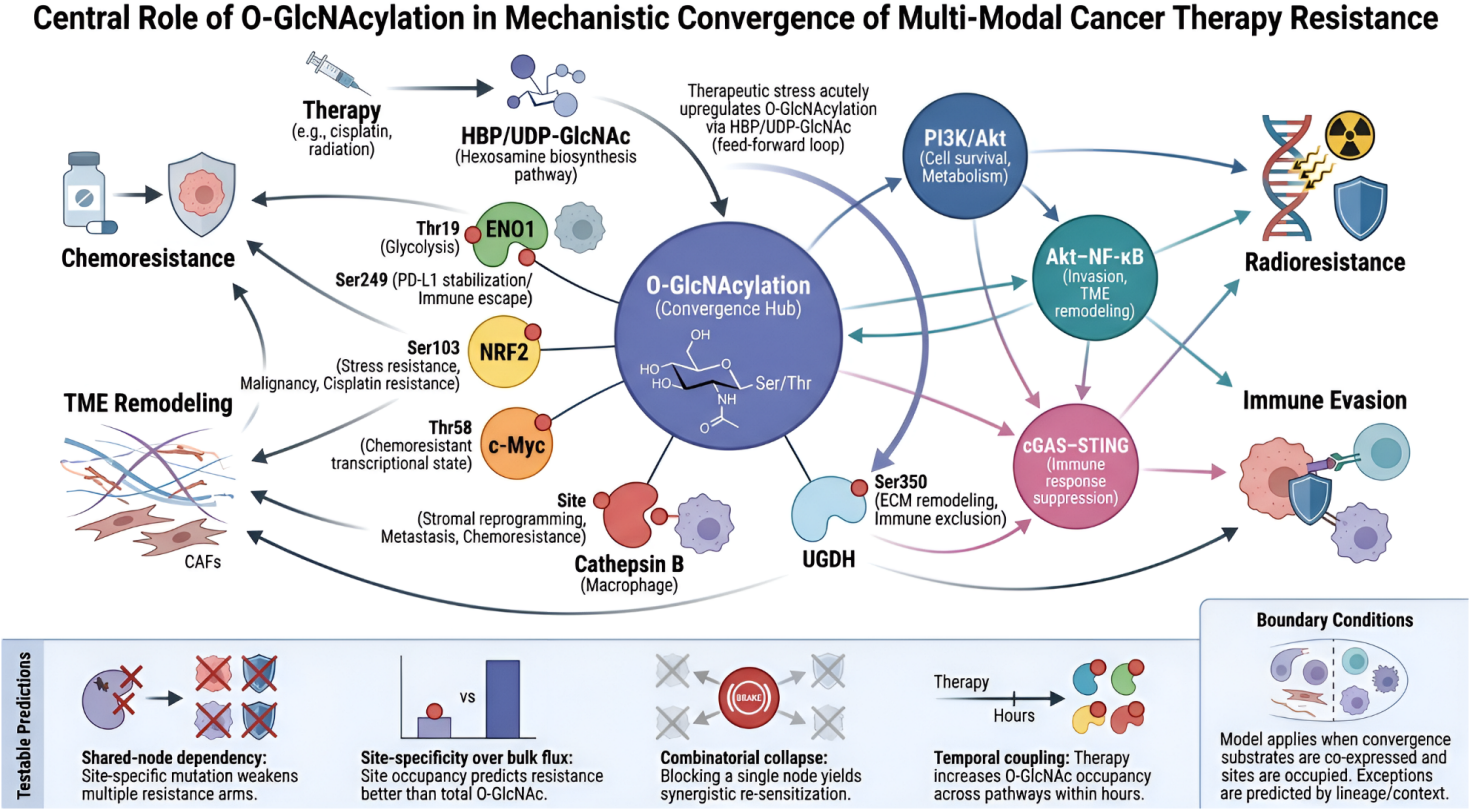
O-GlcNAcylation as a central convergence hub underlying multi-modal cancer therapy resistance. Therapeutic stressors, including chemotherapy and radiotherapy, acutely enhance flux through the hexosamine biosynthetic pathway (HBP), increasing UDP-GlcNAc availability and global O-GlcNAcylation. Elevated O-GlcNAcylation functions as a convergence hub that integrates diverse resistance programs through shared substrates and signaling pathways. At the substrate level, site-specific O-GlcNAc modifications of ENO1, NRF2, c-Myc, Cathepsin B, and UGDH couple metabolic adaptation, stress resistance, stromal remodeling, extracellular matrix reprogramming, and immune exclusion across multiple resistance arms. At the pathway level, O-GlcNAcylation converges on common signaling hubs, including PI3K/Akt, Akt–NF-κB, and cGAS–STING, thereby coordinating cell survival, invasion, tumor microenvironment (TME) remodeling, immune suppression, and radioresistance. Collectively, these interactions link the four canonical manifestations of therapeutic failure—chemoresistance, radioresistance, immune evasion, and TME remodeling—into an interconnected network of cross-resistance. The lower panel summarizes key experimentally testable predictions of the convergence model: (i) shared-node dependency, whereby disruption of a single O-GlcNAc-regulated substrate weakens multiple resistance arms; (ii) site-specificity over bulk flux, in which substrate occupancy predicts resistance more accurately than total O-GlcNAc abundance; (iii) combinatorial collapse, whereby inhibition of a shared convergence hub produces supra-additive re-sensitization across therapeutic modalities; and (iv) temporal coupling, wherein therapeutic stress rapidly induces O-GlcNAc occupancy changes across pathways traditionally assigned to distinct resistance programs. The model is bounded by substrate co-expression and site occupancy, defining the biological context in which convergence is expected to occur.

To qualify as a mechanistic framework rather than a descriptive label, the convergence model must make predictions that differ from the two prevailing alternatives: the independent-arms view, in which chemoresistance, radioresistance, immune evasion, and TME remodeling evolve through separately wired effectors, and the bulk-flux view, in which malignancy scales with global OGT activity or total O-GlcNAc abundance. The convergence model differs from both in asserting that a limited set of shared, site-specific substrates and hubs, not global flux and not independent programs, causally links the resistance arms. This yields four falsifiable predictions: First, shared-node dependency: site-specific disruption of an individual convergence substrate (e.g., a non-glycosylatable ENO1-Ser249, NRF2-Ser103, or c-Myc-Thr58 knock-in) should weaken multiple resistance arms simultaneously, whereas the independent-arms model predicts a single-arm effect. Strictly observing modality-restricted phenotypes across well-powered assays would falsify convergence. Second, site-specificity over bulk: substrate-resolved O-GlcNAc occupancy at these nodes should predict multi-modal resistance better than total O-GlcNAc or OGT levels; if the bulk signal consistently outperforms site-resolved occupancy, the bulk-flux model is favored. Third, combinatorial collapse: Because the arms share nodes, blocking one convergence hub should produce supra-additive re-sensitization across modalities, whereas independently wired resistance predicts additive effects at best. Fourth, temporal coupling: a therapeutic stress applied to one arm should acutely elevate O-GlcNAc occupancy on substrates nominally assigned to the others within hours; the absence of such cross-arm occupancy changes would argue against a shared temporal hub. Critically, the model is bounded rather than universal; it predicts convergence only when substrates are co-expressed and relevant sites are occupied. Therefore, lineage-specific or directionally reversed outcomes (such as the stage-dependent reversal of OGT effects on 5-FU sensitivity (123)) are not exceptions to be explained away, but boundary conditions that the model explicitly anticipates. Distinguishing genuine convergence from coincidental co-regulation will require site-resolved multi-arm perturbation in isogenic systems, an experimental program that descriptive literature has not yet undertaken (**Figure 5**).

### 7.2 The O-GlcNAc–phosphorylation yin-yang as a dynamic resistance switch

A distinct but complementary layer of regulation within the convergence model is the site-specific competition between O-GlcNAcylation and Phos. Because these two modifications frequently target identical or proximal serine/threonine residues, their reciprocal occupancy constitutes a dynamic switch that enables malignant cells to reconfigure their proteome within minutes of therapeutic insult and coordinate multiple resistance arms through a single post-translational code (44, 124). The most direct embodiment of this reciprocal regulation operates on the androgen receptor in castration-resistant prostate cancer, where lncRNA LINC01126 orchestrates a literal switch from O-GlcNAcylation at Thr80 to phosphorylation at Ser81 by recruiting CDK9 and displacing OGT, thereby sustaining ligand-independent AR transactivation (124). An inverse yet conceptually identical mechanism operates on RSK4 in esophageal squamous cell carcinoma, where O-GlcNAcylation at Thr405 sterically antagonizes GSK3β-mediated phosphorylation at the adjacent Thr402/Ser406, preventing FBXW7-dependent degradation and entrenching cancer stem-cell traits and radioresistance. Conversely, in breast cancer, ULK1-driven phosphorylation of PKM2 at Ser333 displaces its O-GlcNAc modification, enforcing tetramerization, suppressing nuclear c-Myc signaling, and restraining the Warburg effect (44), demonstrating the bidirectional, mutually antagonistic nature of this interplay. This yin-yang dynamic enables rapid, reversible transitions between therapy-sensitive and therapy-resistant states, permitting tumors to fine-tune metabolic reprogramming, hormone receptor activity, and stress-adaptive signaling on timescales far shorter than those allowed by transcriptional or genetic adaptation.

## 8 Targeting O-GlcNAcylation: therapeutic strategies and future perspectives

### 8.1 Pharmacological modulation of O-GlcNAcylation: from catalytic inhibition to substrate-directed platforms

**Figure 6:**
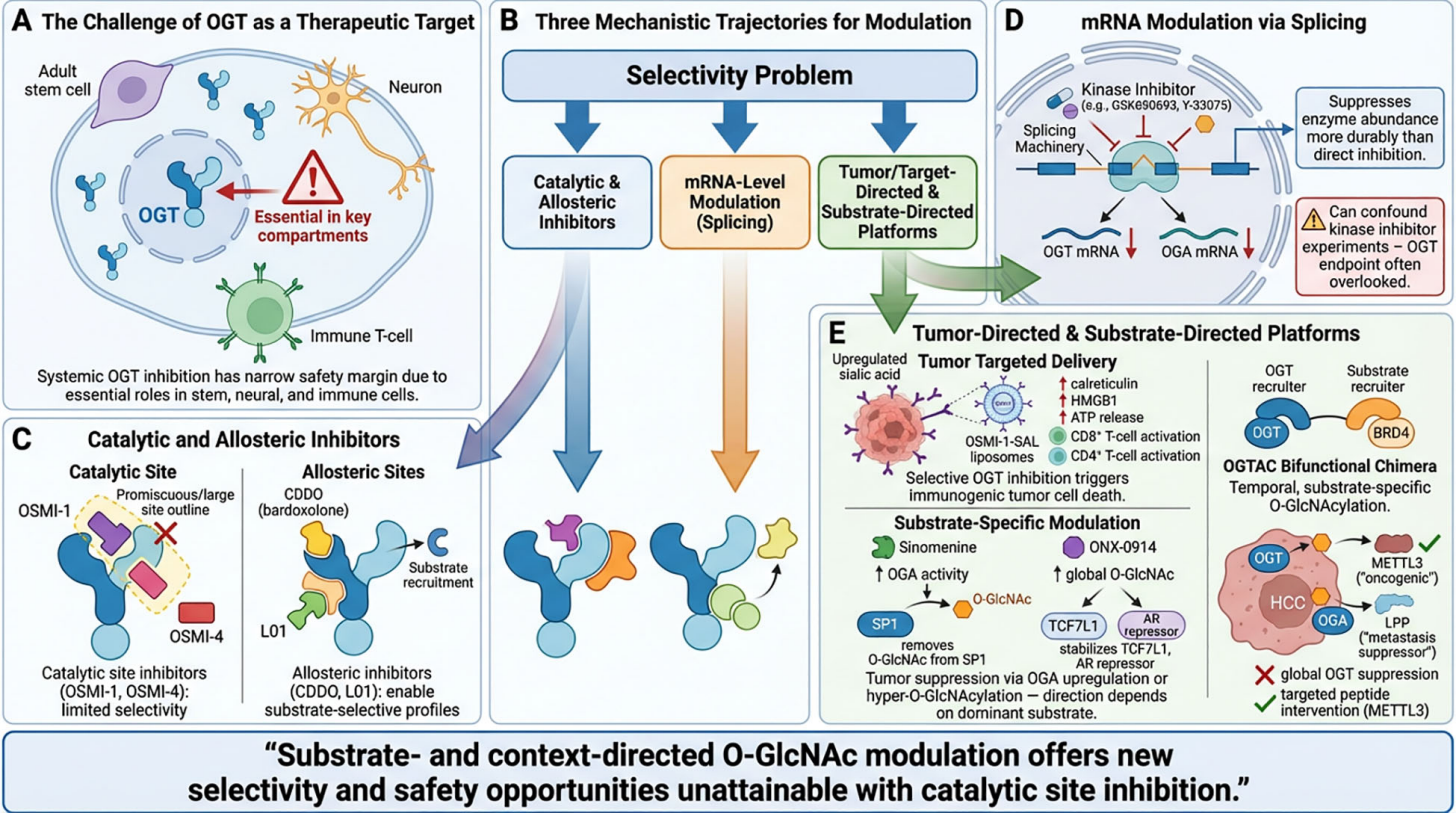
Substrate-selective O-GlcNAcylation circuits in cancer drug resistance. **(A)** O-GlcNAcylation contributes to drug resistance through regulation of DNA repair, transcriptional programs, and metabolic reprogramming in cancer cells. **(B)** Overview of context-dependent O-GlcNAc signaling circuits and their role in therapeutic resistance and tumor adaptation. **(C)** Mechanistic diversity of O-GlcNAc regulation in cancer biology, highlighting substrate-specific effects on oncogenic pathways. **(D)** mRNA-level and splicing-based regulation of OGT/OGA expression influencing global O-GlcNAcylation levels and downstream resistance phenotypes. **(E)** Tumor- and substrate-directed therapeutic strategies, including targeted delivery systems and bifunctional OGT-based platforms, enabling selective modulation of O-GlcNAc signaling.

The central therapeutic challenge posed by OGT is that its essentiality in adult stem cells, neurons, and immune compartments renders systemic catalytic inhibition an inherently narrow-window strategy. This narrow window is most acute in the central nervous system, where the consequences of O-GlcNAc depletion are both severe and directionally opposite to those sought in cancer therapy. Forebrain-specific OGT deletion in adult mice precipitates progressive neurodegeneration, including neuronal loss, gliosis, accumulation of hyperphosphorylated tau and amyloidogenic Aβ peptides, and memory impairment, establishing that sustained neuronal hypo-O-GlcNAcylation is a degenerative insult (125). Consistent with this, germline hypomorphic OGT variants in humans cause an X-linked congenital disorder of glycosylation characterized by intellectual disability and developmental delay (126), indicating that even a partial loss of OGT function is poorly tolerated by the human brain. The directional concern is sharpened by the prevailing CNS therapeutic strategy, which moves in the opposite direction: because O-GlcNAcylation of tau antagonizes its phosphorylation and aggregation, raising brain O-GlcNAc through OGA inhibition is neuroprotective (127), and underlies the brain-penetrant OGA inhibitors now in clinical development for Alzheimer’s disease and progressive supranuclear palsy (e.g., LY3372689, BIIB113, and FNP-223) (128–131). Therefore, a systemically administered CNS-penetrant OGT inhibitor would be expected to exert inverse pharmacology, lowering neuronal O-GlcNAc and potentially promoting tau hyperphosphorylation (127), synaptic dysfunction, and feeding and metabolic dysregulation associated with hypothalamic OGT loss (132). Thus, blood–brain barrier permeability becomes a defining design axis rather than a peripheral pharmacokinetic detail: for the many extracranial malignancies discussed here, a deliberately BBB-excluded, peripherally restricted OGT inhibitor would be desirable to spare the CNS, whereas for intracranial tumors such as glioblastoma, in which OGT-dependent circuits are themselves implicated (42, 115), the BBB penetration required for efficacy places the drug precisely where neuronal O-GlcNAc depletion is most hazardous. These considerations suggest that any clinical program involving OGT inhibition should incorporate an explicit characterization of brain exposure along with prospective neurocognitive pharmacovigilance. They provide an additional safety-driven rationale for tumor-restricted delivery and substrate-directed strategies, which confine O-GlcNAc modulation to the malignant compartment and away from vulnerable neurons. These mechanism-defined strategies set the stage for the rational drug combinations and biomarker-guided regimens discussed below (**Table 5**), and their relationship with the convergence framework is summarized in **Figure 6**. The field has evolved along several conceptually distinct trajectories, each addressing the selectivity problem from a different mechanistic perspective (**Table 6**).

**Table 5:** Therapeutic targeting of O-GlcNAcylation in cancer: small-molecule inhibitors, combination strategies, and predictive biomarkers.

Cancer model/cohort	Therapeutic agent/strategy	Regulator(s)/mechanism of O-GlcNAc modulation	O-GlcNAc target/downstream pathway	Key findings	Evidence level	Ref.
HCC (*in vitro* & *in vivo*; 50 paired tumors + TCGA)	SA-modified OSMI-1 liposome (OSMI-1-SAL)	OGT inhibition; targeted delivery via sialic-acid liposome	Tumor-cell OGT; CD8+/CD4+ T-cell infiltration; Treg	ICD + antitumor T-cell immunity; OGT correlates with prognosis	Human/clinical	(133)
HCC cells; *in vivo*	Sinomenine (SIN) — natural product	Upregulates OGA → reduces O-GlcNAc	SP1 (Thr407)	OGA↑ → SP1-T407↓; suppresses HCC growth	Mouse *in vivo*	(134)
Hormone-sensitive prostate cancer (HSPC); CRPC; xenograft	ONX-0914 (immunoproteasome inhibitor)	HBP activation → global O-GlcNAcylation↑ (LMP7-dependent and -independent)	TCF7L1 → AR repression	TCF7L1 stabilization, AR repression; tumor growth *in vivo*	Mouse *in vivo*	(135)
Cell-based (BRD4, CK2α, EZH2)	OGTACs — heterobifunctional small molecules	Recruit FKBP12F36V-fused OGT to target proteins	BRD4, CK2α, EZH2 (protein-specific O-GlcNAc)	Protein-specific O-GlcNAcylation tool in living cells	*In vitro*	(136)
Mantle cell lymphoma (BTZ-resistant lines; primary patient cells)	Bortezomib (BTZ) + ketoconazole (KCZ, OGA inhibitor)	OGA inhibition (KCZ); CRISPRi MGEA5	Truncated Bid (tBid)	tBid stabilization; sensitizes BTZ-resistant MCL	Human/clinical	(137)
Cell-based screens (drug repurposing, *in vitro*)	GSK690693, Y-33075, OTS964, indisulam, GNF2133	Splicing modulation simultaneously downregulating OGT and OGA (independent of AKT/ROCK)	Global O-GlcNAc homeostasis	Disrupt O-GlcNAc cycling	*In vitro*	(138)
HTRF biochemical & cell-based	CDDO (Bardoxolone)	Direct OGT binding (Kd ≈1.7 µM; IC50 6.56 µM); binds TPR/N-terminal domain	OGT (non-catalytic site)	Novel OGT inhibitor (distinct pocket)	*In vitro*	(139)
HepG2 HCC; xenograft	Doxorubicin (DOX) + OSMI-1	OGT inhibition	p53/mitochondrial Bcl-2	p53/Bcl-2 apoptosis; tumor growth↓ *in vivo*	Mouse *in vivo*	(92)
Cellular & zebrafish	Natural product L01	Structure-based virtual screening; binds Asn557 near UDP pocket	OGT	OGT inhibitor; low zebrafish toxicity	*In vitro*	(140)
Mammalian cell lines	OSMI-1	High-throughput screening hit	OGT (cell-permeable inhibitor)	First cell-permeable OGT inhibitor	*In vitro*	(141)
Prostate & breast cancer xenografts	Glycopeptide-based PKM2 nano-activator (OGA-activated)	OGA-cleavable nanoassembly → *in situ* PKM2 tetramerization	PKM2 (Domain C, dimer→tetramer)	Resensitizes tumors to chemo *in vivo*	Mouse *in vivo*	(142)
NSCLC cell lines, organoids, mouse models	Mannose (oral) ± immune-checkpoint inhibitor	Directly targets OGT → suppresses hnRNP R O-GlcNAcylation; modulates gut microbiota	hnRNP R → JUN mRNA stability → IL-8	OGT/hnRNP R/JUN/IL-8 axis; enhances ICI	Mouse *in vivo*	(143)
Human cancer datasets; KrasG12D PDAC mouse line	ERK inhibition (mechanistic)	OGA/OGT mutual transcriptional regulation via p300 + C/EBPβ; ERK signaling	O-GlcNAc homeostasis	OGA/OGT transcriptional loop in PDAC	Human/clinical	(144)
Cholangiocarcinoma (CCA) cells (KKU-213/214 ± metastatic L5)	siOGT; PSA lectin	OGT silencing	FOXO3 → MAN1A1 → Hex9HexNAc2 high-mannose & biantennary N-glycans	FOXO3/MAN1A1 → high-mannose N-glycans	*In vitro*	(145)
CCA cell lines	siOGT/siOGA (mechanistic)	OGT modulation	NF-κB (O-GlcNAc-modified) → MMPs	NF-κB O-GlcNAc → MMPs	*In vitro*	(146)
Breast cancer cells; human tumors	OGT/OGA modulation (mechanistic)	HBP/OGT	HIF-1α → GLUT1; CHOP/ER stress	HIF-1α/GLUT1; poor outcome correlation	Human/clinical	(20)
Mass-forming CCA tissues (IHC)	Biomarker study	OGT↑, OGA↓, OGP↑	O-GlcNAc/OGT/OGA	OGT↑/OGA↓ correlate with poor survival	Human/clinical	(147)
Neuroblastoma (158 tumors; MYCN-amplified lines; Th-MYCN mice)	Thiamet G (OGA inhibitor)	OGA inhibition → O-GlcNAc accumulation	GSK3β (Ser9) → MYCN (Thr58) degradation	GSK3β→MYCN degradation; high O-GlcNAc predicts favorable prognosis	Human/clinical	(148)
216 NSCLC patients receiving radiotherapy	Radiotherapy (predictive biomarker)	Pretreatment IHC of tumor O-GlcNAc	O-GlcNAc level	Low O-GlcNAc → poor RT response (OR 2.47)	Human/clinical	(117)
278 breast cancer cases (IHC cohort)	Prognostic biomarker	High cytoplasmic & nuclear O-GlcNAc	HAS1-3 → hyaluronan accumulation	High O-GlcNAc → relapse/metastasis/poor outcome	Human/clinical	(149)
109 osteosarcoma patients (Enneking IIB/III)	Prognostic biomarker (chemotherapy response)	OGA expression (IHC)	OGA	Low OGA → poor survival & chemo response	Human/clinical	(150)
HR+/HER2− luminal breast cancer (METABRIC + IHC cohort)	Recurrence prognostic biomarker	O-GlcNAc + PKM2 IHC	O-GlcNAc & PKM2	High O-GlcNAc/PKM2 → poor 10-yr DFS	Human/clinical	(151)
HCC (40 LT tumors; 60 HCC samples; cell lines)	Liver-transplantation recurrence biomarker	OGT/OGA mRNA & global O-GlcNAc	E-cadherin, MMP1/2/3	High O-GlcNAc/low OGA → recurrence (HR 0.438)	Human/clinical	(152)
Endometrial cancer (219 tumors + TCGA; PDOs; mouse)	Stratification & therapeutic vulnerability	FBXO31 (E3 ligase) ubiquitinates OGT	OGT (FBXO31-mediated ubiquitination)	FBXO31 ubiquitinates OGT; high O-GlcNAc tracks grade	Human/clinical	(153)
KRAS-mutant PDAC (PDX, PDOs, KPC, KPC-Cldn18.2 KO mice)	MRTX1133 (KRASG12D inhibitor) + anti-CLDN18.2	KRAS mutation + hyperglycemia → O-GlcNAcylation	CLDN18.2 (Thr204) → PTP1B/Src	CLDN18.2-T204 O-GlcNAc → therapy resistance	Human/clinical	(154)
HCC (*in vitro*, *in vivo*, tail-vein metastasis)	Mechanistic/therapeutic concept	TGF-β1 → GFAT1 → HBP/OGT; UBE3A	LPP (Ser33/Ser35; K108 ubiquitination)	TGF-β1→GFAT1→LPP O-GlcNAc; metastasis	Mouse *in vivo*	(155)
HCC (*in vitro* & *in vivo*)	Designed inhibitory peptides	O-GlcNAcylation of METTL3 (T186/S192/S193) reduces ubiquitination; enhances WTAP binding	METTL3 → m6A-IGF2BP3 → MCM10	METTL3 O-GlcNAc → m6A-MCM10; peptides inhibit HCC	Mouse *in vivo*	(156)
Melanoma cell lines (MNT-1, SK-MEL-28, A-375); GEO data	Mechanistic	siOGT	Akt → NF-κB → ZEB-2, MCT-1	Akt-NF-κB → migration/invasion	*In vitro*	(122)
Breast cancer (palbociclib-resistant cells, PDX, patients)	CDK4/6 inhibitor (palbociclib) + PDGFR inhibitor	OGT	RUNX1 (Ser252) → PDGF-BB → Akt; senescence suppression	RUNX1-S252 O-GlcNAc → resistance	Human/clinical	(157)
Breast cancer (palbociclib-resistant cells, PDX, patients)	CDK4/6 inhibitor (palbociclib) + MITF/OGT inhibition	OGT	MITF (Ser49) → importin α/β → nuclear import; senescence suppression	MITF-S49 O-GlcNAc → resistance	Human/clinical	(158)

Evidence level = highest validation reached: *In vitro* (cells only); Mouse *in vivo* (animal models, ± confirmatory human staining); Human/clinical (patient cohorts with survival/prognosis/treatment-response correlation).

**Table 6:** Intervention strategies for modulating O-GlcNAcylation in cancer, organized by therapeutic mechanism.

Strategy class	Representative agent(s)	Mechanism of intervention	Selectivity/therapeutic-window advantage	Highest evidence level	Ref.
Catalytic-site inhibition	OSMI-1; OSMI-4	Block UDP-GlcNAc transfer at the OGT active site	Proof-of-concept; limited by large promiscuous active site	Mouse *in vivo* (xenograft)	(141, 159)
Allosteric inhibition	CDDO (bardoxolone); L01	Disrupt substrate recruitment (TPR/N-terminal) or UDP-pocket-adjacent surface (Asn557)	Substrate-selective profiles unattainable by catalytic blockers	*In vitro*/biochemical	(139, 140)
mRNA-level/splicing modulation	GSK690693, Y-33075, OTS964, indisulam, GNF2133	Splicing-mediated coordinate downregulation of OGT/OGA abundance	Suppresses steady-state enzyme more durably than activity	*In vitro*	(138)
Tumor-directed delivery	OSMI-1–sialic-acid liposomes (OSMI-1-SAL)	Receptor-mediated tumor-selective delivery; triggers immunogenic cell death	Confines O-GlcNAc suppression to tumor; spares normal tissue	Mouse *in vivo* (orthotopic HCC)	(133)
Substrate-directed (writing)	OGTAC chimeras (BRD4, CK2α, EZH2)	Impose site-specific O-GlcNAcylation on defined targets with temporal control	Causal, single-substrate resolution; avoids global pleiotropy	*In vitro* (proof-of-concept)	(136)
Substrate-interface peptides	OGT–GR competitive peptide; HGS glycosylation peptide; METTL3-directed interference	Block O-GlcNAcylation of one substrate via PPI disruption	Site-selective; preserves protective modifications on other substrates	Mouse *in vivo*	(27, 57, 156)
OGA-based (eraser activation/triggers)	Sinomenine (OGA activation); OGA-triggered PKM2 nano-activator; ONX-0914 (O-GlcNAc–elevating)	Remove O-GlcNAc from oncogenic substrate, or exploit tumor OGA as a spatial trigger	Direction tuned to dominant substrate; tumor-context-selective	Mouse *in vivo*	(134, 135, 142)

Evidence level = highest validation reached: *In vitro* (cells only); Mouse *in vivo* (animal models, ± confirmatory human staining); Human/clinical (patient cohorts with survival/prognosis/treatment-response correlation).

#### 8.1.1 Catalytic and allosteric inhibitors of the target protein

The first-generation inhibitor OSMI-1 established proof-of-concept that cell-permeable OGT blockade is achievable without perturbing cell-surface glycans (141), and the structure-guided evolution of the quinolinone-6-sulfonamide scaffold subsequently yielded low-nanomolar agents, such as OSMI-4, with genuine in-cell pharmacology (159). However, these catalytic-site inhibitors remain constrained by OGT’s large, hydrophilic, and promiscuous active site, which has historically resisted true selectivity. The conceptual advance arrived with allosteric engagement: CDDO (bardoxolone), identified through HTRF screening, binds the tetratricopeptide repeat and N-terminal domains with a K_d of ~1.7 μM and, critically, disrupts substrate recruitment rather than sugar transfer, a binding mode unaffected by OSMI-4 (139). The natural product L01, which contacts Asn557 near the UDP-binding pocket, defines a second tractable allosteric surface (140). Together, these agents reframe OGT from a monolithic catalytic target into a multi-surface enzyme whose distinct interaction interfaces may yield substrate-selective inhibition profiles that cannot be achieved by catalytic-site blockers, a shift with direct implications for the therapeutic window problem.

#### 8.1.2 mRNA-level modulation via splicing

An orthogonal strategy emerged unexpectedly from drug-repurposing screens, which revealed that several “kinase inhibitors”, GSK690693, Y-33075, OTS964, indisulam, and GNF2133–act as splicing modulators that coordinately downregulate OGT and OGA mRNA, independently of their annotated targets (138). This finding has two implications that extend beyond the compounds themselves: mechanistically, it demonstrates that steady-state enzyme abundance can be suppressed more durably than catalytic activity; interpretively, it suggests that several published phenotypes attributed to kinase inhibition may be OGT-mediated, a caveat that warrants systematic reevaluation of the kinase-inhibitor literature in which O-GlcNAc endpoints were not measured.

#### 8.1.3 Tumor-directed delivery and substrate-directed platforms

The most clinically proximate solution to the therapeutic index problem is not a better inhibitor but a better delivery or targeting strategy. Sialic acid-decorated liposomes loaded with OSMI-1 (OSMI-1-SAL) confer receptor-mediated tumor selectivity in HCC and, beyond simple cytotoxicity, trigger immunogenic cell death with calreticulin, HMGB1, and ATP release, and intratumoral CD8^+^/CD4^+^ T-cell activation (133), directly addressing the inverse correlation between OGT expression and cytotoxic T-cell infiltration observed in HCC. Two contrarian findings further reshape the inhibitor-centric paradigm: sinomenine suppresses tumor growth not by blocking the writer but by activating the eraser OGA to remove O-GlcNAc from SP1 at Thr407 (134), while ONX-0914 paradoxically suppresses hormone-sensitive prostate cancer by *elevating* global O-GlcNAcylation to stabilize the AR repressor TCF7L1 (135). Together, these agents establish that the therapeutic direction of O-GlcNAc modulation, increase or decrease, depends entirely on which substrate dominates the tumor’s oncogenic logic, not on a universal “OGT = bad” heuristic.

The logical endpoint of this substrate-centric evolution is the OGTAC platform, which comprises bifunctional chimeras that impose site-specific O-GlcNAcylation on defined targets, such as BRD4, CK2α, and EZH2, with temporal control (136). The OGTAC represents the first platform capable of causally dissecting substrate-level O-GlcNAc biology in ways that global inhibitors cannot. Its conceptual extension to tumor-specific contexts is already evident. In HCC, for instance, OGT simultaneously stabilizes the oncogenic m6A methyltransferase METTL3 (156) and degrades the metastasis suppressor LPP (155), two opposing consequences of the same enzyme within the same tumor, nominating peptide-based interference with METTL3 O-GlcNAcylation as a more rational intervention than global OGT suppression, which would simultaneously remove the protective LPP modification. This duality is the clearest single argument against catalytic site inhibition as the endpoint of the field: substrate-directed intervention is not merely an incremental refinement but a conceptual necessity. Importantly, the common concern that OGT modifies thousands of substrates applies to global catalytic inhibition and not to OGTAC. By recruiting OGT only to a predefined target through a bifunctional chimera, OGTAC is designed to confine O-GlcNAcylation to a single substrate with inducible, dose-tunable, and reversible control, thereby minimizing the pleiotropy that limits active-site inhibitors (136). This selectivity is nonetheless a design goal rather than a proven property: residual proximity-driven modification of neighboring proteins, unintended downstream consequences of forced modification of the target itself, and the fact that OGTAC remains an early, *in vitro* proof-of-concept lacking *in vivo* efficacy, pharmacokinetic, tumor-delivery, and safety data, all mean that its therapeutic index must be established empirically before it can be considered an ideal intervention. Therefore, we present OGTAC not as a finished solution but as the most conceptually rational template for substrate-directed intervention, whose off-target profile and translatability remain priority questions for the experimental program proposed below.

The translational priorities that follow from this analysis are as follows: co-crystal structures of CDDO– and L01–OGT complexes to enable rational allosteric design; systematic substrate proteomics under each inhibitor class to map selectivity landscapes; and combination of OSMI-1-SAL with immune-checkpoint blockade in orthotopic models. Whether OGT transitions from a compelling cancer target to a clinically actionable one will depend less on potency gains and more on whether the field commits to substrate-level rather than enzyme-level interventions.

### 8.2 Rational combinations with chemo-, radio-, and immunotherapy

A recurring lesson across preclinical studies is that OGT modulation rarely functions as monotherapy; its clinical value lies in the sensitization of co-administered agents, including metabolic, genotoxic, and immunological agents. Two proof-of-concept studies illustrate the opposite but complementary design principles. A glycopeptide nano-activator, triggered specifically by OGA upregulation in the tumor microenvironment, induces PKM2 tetramerization *in situ*, blocks the nuclear translocation of the oncogenic dimeric isoform, and restores chemosensitivity in prostate and breast cancer models (142). Here, OGA serves not as a therapeutic target but as a tumor-selective trigger, repurposing the enzymatic context for spatially confined drug activation in tumor cells. Conversely, mannose acts upstream of the enzyme, suppressing OGT activity to abolish hnRNP R O-GlcNAcylation, destabilize JUN mRNA, and dampen the IL-8-driven inflammatory microenvironment of NSCLC, an effect that enhances immune checkpoint inhibitor efficacy both *in vitro* and *in vivo* (143). These two strategies together establish that O-GlcNAc modulation is most powerful when engineered to exploit tumor-specific vulnerabilities rather than being deployed as a generic enzyme blockade.

For an immunotherapy-oriented readership, the most consequential convergence is that multiple O-GlcNAc-directed strategies now independently nominate OGT modulation as a checkpoint blockade sensitizer. The mannose–ICI combination (143), OSMI-1-SAL-driven immunogenic cell death in HCC (133), and restoration of cGAS-STING-dependent type I interferon signaling upon OGT inhibition collectively suggest that O-GlcNAc suppression can simultaneously reduce tumor-intrinsic immune evasion and increase antigen availability, a dual mechanism that few other metabolic targets offer (**Table 7**). Beyond checkpoint blockade, the broader preclinical landscape documents synergy with doxorubicin in HCC and prostate cancer (92), oxaliplatin in colorectal cancer through OSMI-1-mediated resensitization, and radiotherapy through HBP-dependent DSB repair suppression (118). A mechanistically distinct but near-term combination opportunity arises in breast cancer, where O-GlcNAcylation converges on CDK4/6-inhibitor resistance through two complementary transcription factor circuits. In one, O-GlcNAcylation of RUNX1 at Ser252 prevents its ubiquitin-mediated degradation and sustains PDGF-BB–driven palbociclib resistance, so that repurposing FDA-approved PDGFR inhibitors re-sensitizes RUNX1-high, CDK4/6i-resistant tumors, one of the few O-GlcNAc-informed combinations immediately testable with existing agents (157). In another study, OGT O-GlcNAcylates the microphthalmia-associated transcription factor (MITF) at Ser49, enhancing its importin-α/β–dependent nuclear translocation and suppressing palbociclib-induced senescence. Inhibiting MITF or its O-GlcNAcylation restores palbociclib sensitivity, and MITF activation was confirmed in tumors from palbociclib-resistant patients (158). The convergence of two independent substrates (RUNX1 and MITF) on the same therapeutic vulnerability, each through O-GlcNAc-dependent stabilization, nuclear import, and senescence evasion, exemplifies the substrate-selective convergence logic central to this review and nominates O-GlcNAc modulation as a rational partner for CDK4/6 inhibitors (**Table 5**). The translational question that the field must now answer is not whether OGT modulation synergizes with existing therapies, as this is now firmly established, but which patients, stratified by OGA overexpression or hexosamine pathway flux, will derive the greatest benefit and whether OGA-triggered and mannose-driven platforms offer sufficient mechanistic advantage over simpler pharmacological combinations to justify their developmental complexity.

**Table 7:** Therapeutic strategies targeting O-GlcNAcylation at the tumor–immune interface, organized by mechanism of action.

Therapeutic strategy/agent	Molecular target (substrate/pathway)	Mechanism and antitumor effect	Cancer type (model)	Evidence level	Rational combination	Ref.
Immune checkpoint (PD-1/PD-L1) axis						
HGS glycosylation competitive peptide inhibitor	HGS/PD-L1	Restores lysosomal degradation of PD-L1 and lowers surface PD-L1, relieving T-cell suppression	Multiple (immune-competent mouse)	Mouse in vivo	Anti-PD-L1 antibody	(27)
GFPT1 silencing	GFPT1/PD-L1	Reduces global O-GlcNAc and destabilizes PD-L1, attenuating immune escape	Breast cancer	Mouse in vivo	Checkpoint blockade	(45)
Tumor-associated macrophage (TAM) reprogramming						
OGT deletion/deficiency in TAMs	OGT/EGR2	Blocks protumoral TAM differentiation; preserves CD8^+^ T-cell cytotoxicity and reduces exhaustion	Mouse tumor models	Mouse in vivo	Checkpoint blockade	(49)
Astragalus polysaccharide (APS)	OGT/global O-GlcNAc	Repolarizes TAMs toward NLRP3^+^ M1-like; boosts CD8^+^ infiltration and CXCL10	Orthotopic HCC	Mouse in vivo	—	(51)
Cytotoxic lymphocyte (NK/CD8^+^ T) enhancement						
Thiamet G; genetic OGT-ISS deletion	OGA inhibition/OGT stabilization	Stabilizes O-GlcNAc, preserving NK cytotoxicity under TME-mimicking suppression; enhances tumor killing	Lymphoma; engineered NK92	Mouse in vivo/engineered cells	Adoptive NK-cell therapy	(52)
D-mannose supplementation	OGT/β-catenin–Tcf7	Preserves CD8^+^ T-cell stemness and restrains exhaustion, improving adoptive-therapy efficacy	Melanoma/adoptive T cells	Mouse in vivo	Ex vivo expansion/ACT	(56)
Regulatory T-cell (Treg) targeting						
Treg-selective Glut3 inhibition/deletion	Glut3/c-Rel O-GlcNAc	Abrogates intratumoral Treg suppression without systemic autoimmunity	Mouse + human data	Mouse in vivo/human correlative	Checkpoint blockade	(55)
Restoring antigen presentation and innate sensing						
Competitive OGT–GR interface peptide	OGT/GR (Ser132)	Restores MHC-I and lowers PD-L1, reinstating CD8^+^ T-cell recognition and killing	Endometrial cancer	Mouse in vivo	—	(57)
OGT inhibition as innate-immune activator	OGT (HCF-1) → cGAS–STING	Triggers genomic-instability-driven type I IFN and CD8^+^ antitumor immunity	Syngeneic & Apc^Min^ CRC	Mouse in vivo	Checkpoint blockade (cold → hot)	(58)
DHODH inhibition	DHODH/NRP1	Restores MHC-II, recruits immune infiltration and overcomes anti-PD-1 resistance	Breast, lung	Mouse in vivo/human prognostic	Anti-PD-1	(59)
GFPT1 silencing (adenosine axis)	GFPT1/CD39 (Ser239)	Destabilizes CD39, lowering immunosuppressive adenosine; boosts CD8^+^ infiltration	Breast cancer	Mouse in vivo	—	(60)
Tumor microenvironment: ECM remodeling and angiogenesis						
Targeting O-GlcNAc-deficient UGDH	UGDH (Ser350)	Lowers hyaluronic acid and restores CXCL10/STAT1-driven T-cell recruitment	Xenograft	Mouse in vivo	Checkpoint blockade	(69)
Kaempferol	OGT/Hsp47	Reduces UDP-GlcNAc, impairing collagen maturation and secretion (ECM barrier)	Colorectal cancer	Mouse in vivo	—	(70)
OGT inhibition (FOXM1 axis)	OGT/VEGF	Impairs HBP-driven VEGF secretion and angiogenesis	Hepatocellular carcinoma	Mouse in vivo	—	(72)
GFAT1 knockout	GFAT1/SerRS (Ser101)	Abrogates intercellular sEV-driven SerRS O-GlcNAc, attenuating neovascularization	Bladder cancer	Mouse in vivo	—	(73)
Systemic-metabolic and mechanotransduction targeting						
OGT knockdown/OSMI-1	OGT/PI3K-AKT-mTOR	Reverses proliferation, invasion and lymph-node metastasis	Oral squamous cell carcinoma	Mouse in vivo/human correlative	Glycemic control	(76)
Glycemic control in diabetic patients	Systemic HBP flux	Reduces M2-like TAM skewing and tumor O-GlcNAc driven by hyperglycemia	Colorectal; OSCC (clinical correlative)	Human correlative	Adjunct to systemic therapy	(29)
Notch1/OGT-targeted therapy	EPHA2–LYN–OGT/NICD1	Blocks mechano-stemness program; MIR31 loss as a patient-stratification biomarker	Recurrent chordoma	Human correlative/mechanistic	MIR31-guided stratification	(77)
Emerging substrate-directed and biomarker-guided platforms						
OGTAC framework	Recruits OGT to a single predefined target	Inducible, dose-tunable, reversible site-specific modification of one substrate	Proof-of-concept	In vitro	PD-1/PD-L1 blockade (proposed)	(136)
O-GlcNAc gene-signature score	34-gene O-GlcNAc signature	Stratifies TME subtypes; low score predicts better prognosis and immunotherapy benefit	Gastric cancer (5 cohorts)	Human (predictive)	Companion diagnostic	(79)

### 8.3 Biomarker-guided precision medicine

The prognostic value of O-GlcNAcylation is not based on correlation but on mechanism. In cholangiocarcinoma, hepatocellular carcinoma, breast cancer, osteosarcoma, and endometrial cancer, elevated O-GlcNAcylated protein expression is associated with aggressive histology, advanced stage, and poor survival (144–147, 149, 152) because the modification is mechanistically upstream of the signaling cascades that execute aggressiveness. OGT-driven O-GlcNAcylation stabilizes HIF-1α by blocking its pVHL-mediated degradation, sustaining GLUT1 expression, and glycolytic reprogramming (20). In contrast, reduced OGA levels independently predict poor outcomes in breast cancer (149). In cholangiocarcinoma, the cascade is resolved to substrate-level detail: O-GlcNAcylation activates Akt/ERK signaling, hyperphosphorylates FOXO3, suppresses MAN1A1, and generates high-mannose N-glycans that drive metastatic migration (145), whereas O-GlcNAc-modified NF-κB amplifies MMP expression through nuclear translocation (146). Therefore, the question that follows is not whether O-GlcNAc is prognostic but how it should be measured. Favorable prognostic associations in neuroblastoma and a subset of NSCLC demonstrate that pan-O-GlcNAc immunohistochemistry cannot distinguish tumor-suppressive from oncogenic substrate modifications, and the interpretation of global IHC signals in isolation systematically misclassifies patients in lineages where tumor-suppressive substrates dominate.

Three studies establish what biomarker-guided O-GlcNAc treatment could look like when implemented with mechanistic specificity. In endometrial cancer, O-GlcNAcylation level correlates with histological grade across a 219-tumor Chinese cohort validated in TCGA, with FBXO31-mediated ubiquitination of OGT identified as the tumor-suppressive homeostatic brake, providing both a stratification marker and a molecular explanation for grade-specific dysregulation (153). In HR +/HER2⁻ luminal breast cancer, combined O-GlcNAc and PKM2 immunohistochemistry, integrated with T stage, improves 10-year disease-free survival prediction substantially beyond either marker alone (151), demonstrating that even pan-O-GlcNAc signals gain predictive power when integrated with a mechanistically linked partner marker. Most therapeutically consequential is the finding that KRAS mutation and hyperglycemia cooperatively drive O-GlcNAcylation of CLDN18.2 at Thr204 in PDAC, sequestering the target antigen cytoplasmically, and that low-dose MRTX1133 restores membrane CLDN18.2 localization and synergizes with antigen-targeted therapy (154), establishing O-GlcNAc status as a predictive biomarker of antibody-based therapeutic failure (**Table 5**).

The translational template that emerges is specific: prospective biomarker-stratified trials in which O-GlcNAc expression is decomposed by substrate identity through site-specific glycoproteomics, rather than measured as a pan-modification IHC signal, should allocate patients to OGT inhibitor-containing regimens. The CLDN18.2/KRAS paradigm in PDAC is the most immediately actionable instance, combining a KRAS^G12D inhibitor with CLDN18.2-targeted therapy in a biomarker-selected population is mechanistically justified and clinically feasible using existing agents. It offers, in effect, a first-generation test case for whether the conceptual framework outlined in this review can be converted into clinical benefits.

## 9 Limitations and unresolved questions

Notwithstanding the conceptual coherence of the framework outlined above, several limitations constrain the current evidence base and should be explicitly acknowledged before clinical translation is pursued in the future. First, a pervasive methodological constraint undermines the interpretation of biomarkers. The overwhelming majority of prognostic associations derive from pan-O-GlcNAc immunohistochemistry with antibodies such as RL2 or CTD110.6, which integrate signals across thousands of substrates and cannot distinguish oncogenic from tumor-suppressive modifications (144–147, 149, 152); CTD110.6 further cross-reacts with terminal β-GlcNAc on complex N-glycans, introducing a specificity confound that bulk readouts cannot resolve (30, 31). The paradoxical favorable prognostic association observed in a retrospective 216-patient NSCLC radiotherapy (117) directly illustrates this problem: in the absence of substrate-resolved readouts, globally elevated O-GlcNAcylation cannot be mechanistically differentiated between metabolic fitness and resistance-effector activity. The field already possesses chemoproteomic tools to address this deficiency, including isotope-coded photocleavable probes for site-specific quantitative profiling (108) and optimized enrichment workflows for O-GlcNAc site assignment (8). However, their systematic deployment in clinical specimens remains aspirational rather than operational.

Second, nearly all convergence model substrates have been characterized in tumor cells, leaving the O-GlcNAc state of tumor-infiltrating immune cells mechanistically undefined. Although glucose- and glutamine-fueled O-GlcNAcylation is known to control T-cell self-renewal and malignancy (63), and OGT inhibition restores antitumor immunity through cGAS–STING reactivation (58) and immunogenic cell death (133, 143), the single-cell-resolved effects of O-GlcNAc modulation on tumor-infiltrating lymphocytes, tumor-associated macrophages, and dendritic cells within human tumors remain largely unknown. Therefore, any framework that considers O-GlcNAcylation as a tumor-intrinsic phenomenon will systematically underestimate its contribution to immunotherapy resistance.

Third, the therapeutic window problem persists despite chemical progress. OGT is essential across stem cell, neuronal, and immune compartments (63), and its depletion in the adult brain is sufficient to drive neurodegeneration (125). Current catalytic-site inhibitors, from first-generation OSMI-1 (141) to low-nanomolar OSMI-4 (115), have yet to achieve either substrate selectivity or controlled central nervous system exposure (discussed under Pharmacological modulation) required for clinical deployment (100). Fourth, virtually all human evidence to date is retrospective (117, 151, 153, 154), with no prospective biomarker-stratified trials of OGT-directed interventions reported.

Fourth, the evidence base of the field is predominantly preclinical, and the levels of evidence should be weighed accordingly. The mechanistic circuits synthesized here overwhelmingly derive from cancer cell lines and mouse models (xenograft, syngeneic, and genetic), with comparatively few corroborated in human tissue or clinical cohorts. Where human validation exists, it is largely correlative: globally elevated O-GlcNAcylation tracks with aggressive histology and poor survival across cholangiocarcinoma, hepatocellular carcinoma, breast cancer, osteosarcoma, and endometrial cancer (144–147, 149, 152); TAM OGT and Cathepsin B levels co-vary with chemotherapy response and prognosis in patient specimens (50); the intratumoral Treg Glut3 dependency is supported by human data (55); the DHODH/NRP1 ratio is prognostic in human breast and lung cancer (59); and UGDH O-GlcNAcylation inversely correlates with CD8^+^ infiltration by CyTOF (69), while the largest dedicated clinical dataset remains a single retrospective 216-patient NSCLC radiotherapy cohort (117). Critically, no OGT- or OGA-directed agent has completed an oncology clinical trial; the only O-GlcNAc-targeting drugs that have reached patients are OGA inhibitors developed for neurodegeneration, which are pharmacologically opposite to the anticancer strategy. Reproducibility is further complicated by directionally discordant findings within single contexts. For example, manipulating O-GlcNAc did not acutely alter cisplatin sensitivity in one NSCLC study (82), yet conferred robust resistance in another through p53/c-Myc-dependent outcomes (16). In addition, acute pharmacological OGA inhibition failed to augment NK-cell cytotoxicity (53), whereas stable genetic O-GlcNAc elevation did (52). These discordances are attributable principally to the distinction between acute pharmacological and stable genetic perturbations and to p53- and lineage-dependent substrate outcomes. This indicates that mechanistic claims should be qualified by their evidence level (**Tables 1**–**6**) and that prospective, substrate-resolved validation in human specimens remains the field’s principal unmet need.

Future studies should therefore prioritize four directions: (i) site-specific O-GlcNAc proteomic profiling of prospective cohorts; Multiple enrichment strategies exist, including lectin affinity, pan-O-GlcNAc antibodies, metabolic labeling, and chemoenzymatic/photocleavable chemoproteomic probes. A head-to-head benchmarking of affinity enrichment methods showed that each captures only a partial, partly non-overlapping subpopulation of the O-GlcNAc proteome, suggesting that combining approaches improves coverage (160). Because O-GlcNAcylation occurs in a strictly site-specific manner (161), we prioritized methods that resolve individual modification sites. Isotope-coded photocleavable and chemoenzymatic chemoproteomic probes (108), together with optimized site-assignment workflows (8), are well-suited for quantitative, site-resolved mapping. These approaches are ideally cross-validated using orthogonal affinity methods to maximize coverage. (ii) Single-cell characterization of O-GlcNAcylation across intratumoral immune compartments, particularly in checkpoint-blockade responders versus non-responders; (iii) maturation of substrate-directed platforms such as the OGTAC framework (136), including systematic profiling of their off-target O-GlcNAcylation, *in vivo* efficacy, and tumor delivery, prior to and alongside testing in immune-competent orthotopic models combined with PD-1/PD-L1 blockade; and (iv) first-in-human biomarker-selected trials applying the CLDN18.2/KRAS paradigm (154) as a template for rational O-GlcNAc-guided combination therapy.

## 10 Conclusion

Over the past five years, O-GlcNAcylation has emerged as an important regulator of the tumor–immune interface. The studies synthesized here challenge the idea of a single “OGT–cancer axis” and instead point to a network of parallel, substrate-selective circuits shaped by tumor lineage, immune context, and coexisting oncogenic lesions. Three overarching principles can be derived from this study.

First, O-GlcNAcylation plays a prominent role in immune evasion. It intersects multiple nodes of antitumor immunity, including PD-L1 stabilization, reprogramming of tumor-associated macrophages, modulation of NK and T cell function, and attenuation of cGAS–STING signaling, thereby linking the cellular metabolic state to immune privilege. Second, this modification may represent a promising therapeutic opportunity, particularly in the context of resistance to immunotherapy and genotoxic therapy. The rapid, self-amplifying hexosamine flux triggered by therapeutic stress positions O-GlcNAcylation as a potential convergence point of cross-resistance, providing a rationale for exploring its combination with checkpoint inhibitors, chemotherapy, or radiotherapy. Third, the pleiotropy of OGT remains a major translational challenge. Given its essential functions in multiple cell types, non-selective inhibition poses significant risks. Future progress will likely require more selective approaches, such as targeting rate-limiting enzymes in the hexosamine pathway, disrupting specific protein–protein interactions, or developing tissue-restricted degraders, ideally guided by substrate-resolved biomarkers to minimize off-target effects.

However, several key questions remain to be addressed. Does the O-GlcNAc status of tumor-infiltrating immune cells, in addition to tumor cells, influence responsiveness to checkpoint blockade therapy? Can site-specific or substrate-selective modulation recapitulate the beneficial immunological effects while sparing host immunity? Answering these questions will necessitate integrated approaches, including site-resolved glycoproteomics, spatial metabolomics, and physiologically relevant immune-competent models. If the past decade has established O-GlcNAcylation as a contributor to cancer progression, the coming years will determine whether this pathway can be translated into clinically viable and immunologically informed therapies.
